# Supplementary material for: Measuring the Viscosity of the Escherichia coli Plasma Membrane Using Molecular Rotors
Source: Biophys J. 2016 Oct 4;111(7):1528–40. doi: 10.1016/j.bpj.2016.08.020 (PMC5052448; doi:10.1016/j.bpj.2016.08.020)
Supplement: Document S1. Figs. S1–S9 and Table S1 [file mmc1.pdf]

Biophysical Journal, Volume *111*

Supplemental Information

Measuring the Viscosity of the *Escherichia coli* Plasma Membrane Using Molecular Rotors

Jacek T. Mika, Alexander J. Thompson, Michael R. Dent, Nicholas J. Brooks, Jan Michiels, Johan Hofkens, and Marina K. Kuimova

SUPPLEMENTARY MATERIAL

Measuring the viscosity of the *Escherichia coli* plasma membrane using molecular rotors.

JTM[1,#,*], AJT[2,#], MRD[2], NJB[2], JM[3], JH[1], MKK[2,*]

[#] These authors contributed to this work equally.
*To whom correspondence should be addressed: MKK, JTM

Supplementary Figures

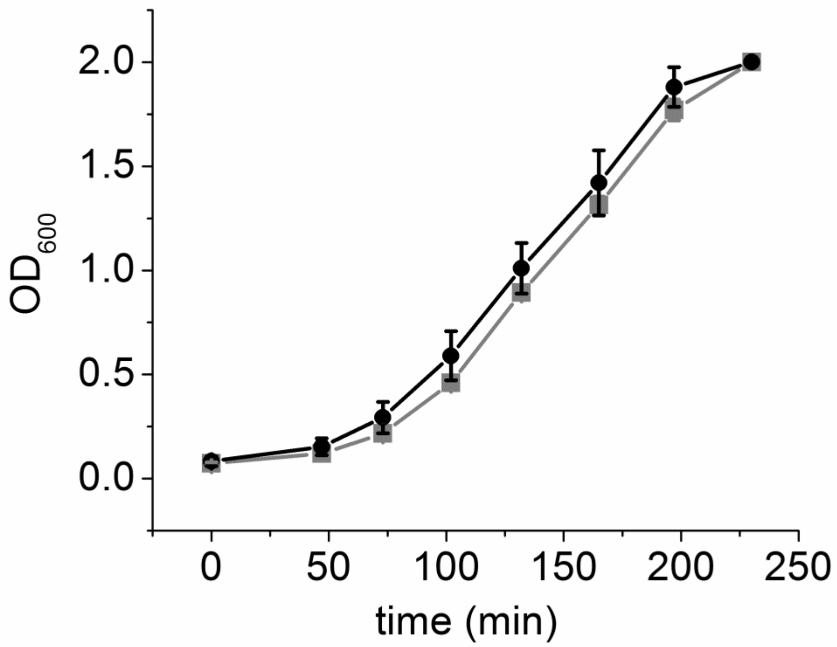

Figure S1. BODIPY C10 does not influence the growth of *Escherichia coli* at the concentrations used for fluorescence lifetime imaging.

E. coli cells were grown in LB medium at 37°C with vigorous shaking (200 rpm). A dense, overnight culture was used to inoculate fresh medium and this was allowed to grow until OD_{600} of 0.1 was reached. The cells grown in the presence of 0.2 µM BODIPY C10 (black circles) had very similar doubling times to cells grown without the dye (grey squares), as monitored by following the optical density of the cultures at 600 nm (OD_{600}). Error bars indicate the standard deviations from three measurements.

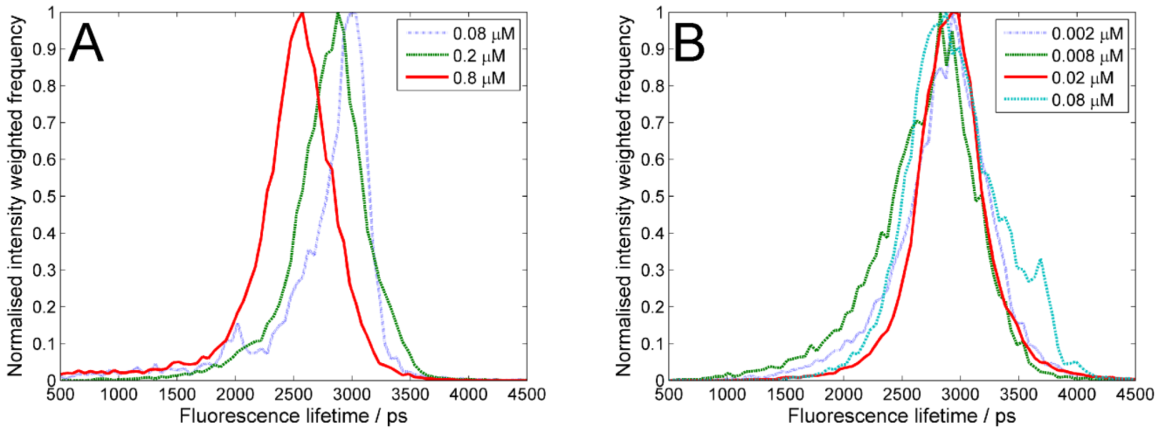

Figure S2. Optimisation of BODIPY C10 concentration to avoid aggregation.

Normalised intensity weighted fluorescence lifetime histograms recorded in populations of (A) healthy cells and (B) spheroplasts using a range of BODIPY C10 concentrations (see keys). Histograms were obtained from images fitted using a mono-exponential decay model (e.g. see Fig. 2). In spheroplasts (B), all lifetime distributions overlap indicating that dye aggregation has no adverse effects on the spheroplast data at any of the concentrations shown. In healthy cells (A), the lifetime distribution observed at a BODIPY C10 concentration of 0.8 μM is lower than that recorded at both 0.2 μM and 0.08 μM. This indicates that aggregation is inducing a reduction in the lifetime of BODIPY C10 at this concentration and, hence, destroying the viscosity sensitivity. At the two lower concentrations (0.2 μM and 0.08 μM) the lifetime distributions in live cells overlap well – although the peaks do not perfectly coincide, the intensity weighted mean fluorescence lifetimes extracted from the two histograms are in agreement to within 50 ps (2740 ps at 0.08 μM and 2790 ps at 0.2 μM). This confirms that aggregation has no impact on the healthy cell data for concentrations at or below 0.2 μM.

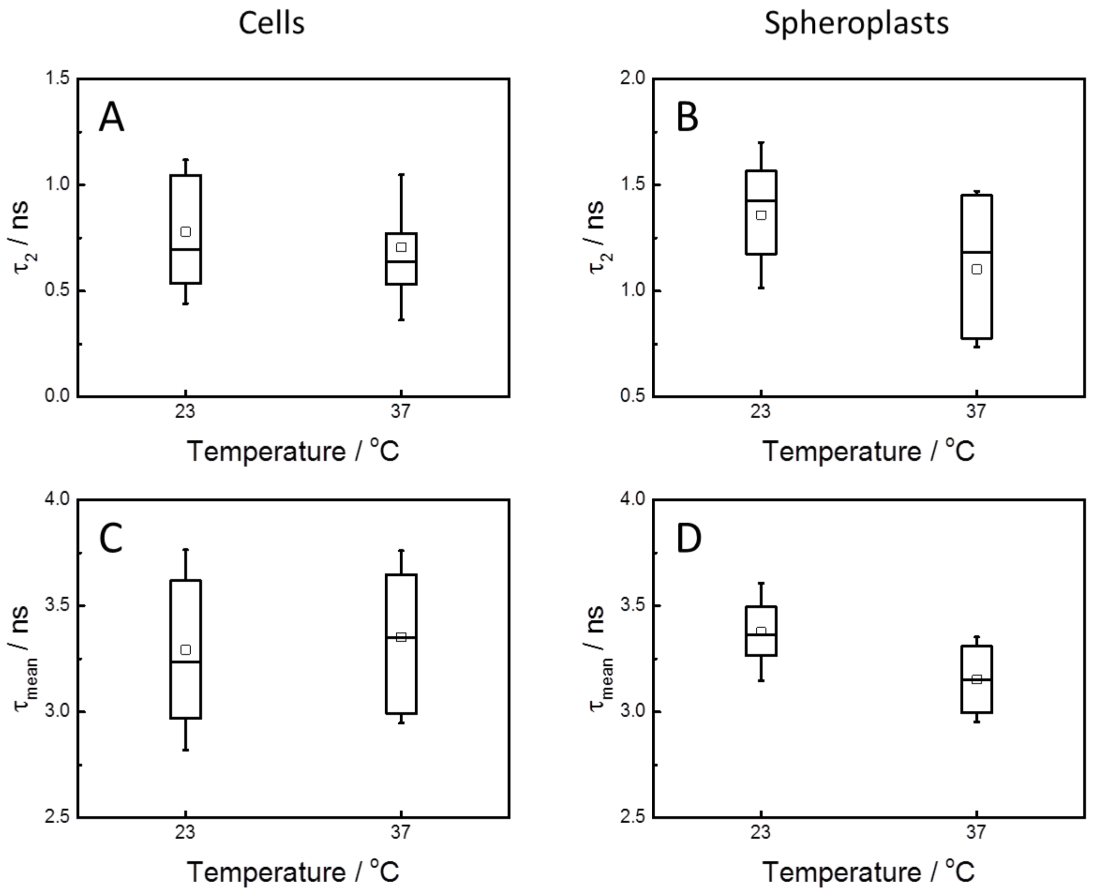

Figure S3. Behaviour of the additional fitted lifetime parameters (τ_2 and τ_{mean}) of BODIPY C10 in the membranes of live *E. coli* cells and spheroplasts.

Box plots showing the behaviour of τ_2 (top row – A, B) and τ_{mean} (bottom row – C, D) in healthy cells (left column – A, C) and spheroplasts (right column – B, D) at both 23° C and 37° C. Boxes represent the interquartile range; error bars (whiskers) indicate one standard deviation from the mean; horizontal lines show the median values; open squares indicate the mean values. Number of measurements: cells at 23° C – 21 cells from two independent experiments; cells at 37° C – 57 cells from four independent experiments; spheroplasts at 23° C – 28 spheroplasts from two independent experiments; spheroplasts at 37° C – 58 spheroplasts from four independent experiments.

Cells

	Lifetime (ns)		Viscosity (cP)	
	23° C	37° C	23° C	37° C
Mean	4.28	3.99	1160	950
SD	1.00	0.58	670	320
Median	4.00	3.78	960	830
1st Quartile	3.67	3.56	770	730
3rd Quartile	4.81	4.28	1410	1090
IQR	1.15	0.72	630	360

Spheroplasts

	Lifetime (ns)		Viscosity (cP)	
	23° C	37° C	23° C	37° C
Mean	4.45	4.06	1200	980
SD	0.42	0.42	240	210
Median	4.51	4.08	1220	980
1st Quartile	4.13	3.70	1010	790
3rd Quartile	4.74	4.43	1360	1170
IQR	0.61	0.73	350	380

Table S1. Statistics of the FLIM measurement of BODIPY C10 in a population of *Escherichia coli* cells and spheroplasts.

Table shows the lifetimes and viscosities observed in *E. coli* cells (top) and spheroplasts (bottom) at both 23° C and 37° C. SD – standard deviation, IQR – inter-quartile range. Number of measurements: n = 57 cells at 37° C; n = 21 cells at 23° C; n = 58 spheroplasts at 37° C; n = 28 spheroplasts at 23° C.

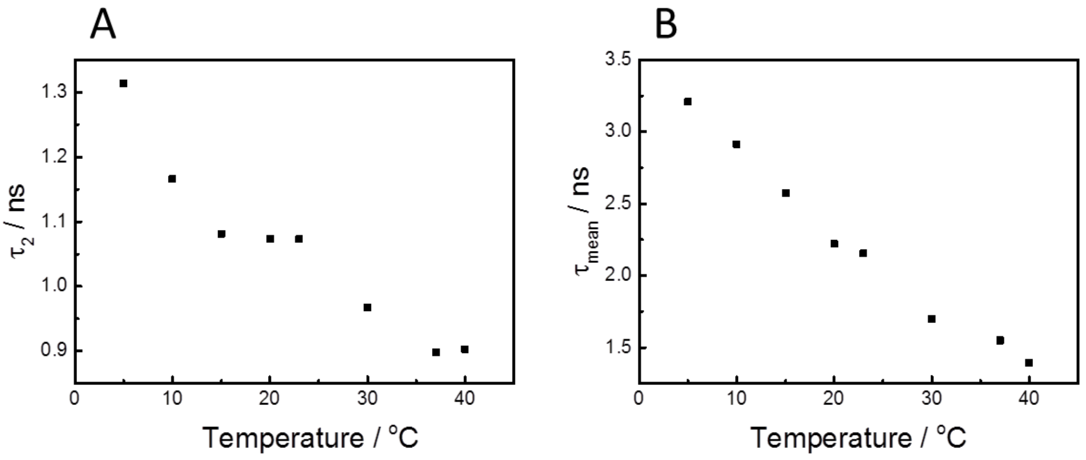

Figure S4. Behaviour of the additional fitted lifetime parameters (τ_2 and τ_{mean}) of BODIPY C10 in liposomes made of *E. coli* lipid extracts as functions of temperature.

TCSPC measurement of BODIPY C10 labelled liposomes composed of *E. coli* membrane lipid extracts, measured as a function of temperature. The behaviour of both the second (short) lifetime component (τ_2 – A) and the weighted mean lifetime (τ_{mean} – B) with respect to temperature are shown. Liposomes were prepared using lipid extracts from *E. coli* cells grown at 37° C.

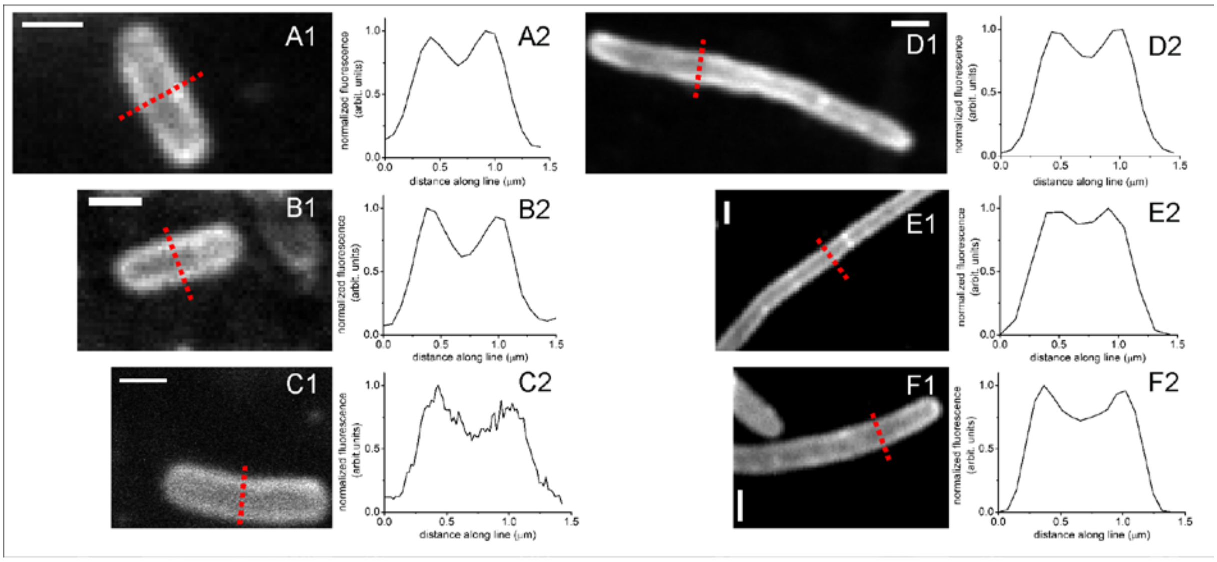

Figure S5. The molecular rotor BODIPY C10 stains membranes in live *Escherichia coli* cells.

Confocal fluorescence images of healthy (A1-C1) and cephalexin treated (D1-F1) *E. coli* cells stained with BODIPY C10. Scale bars 1 μm. Line profiles of fluorescence intensity were extracted along the red dotted lines shown in each image. The line profiles (subpanels with index 2, A2-F2) have two maxima that correspond to the locations of the membranes in the fluorescence images. Staining and fluorescence profiles of this sort indicate that the fluorescent probe BODIPY C10 is located within the membrane.

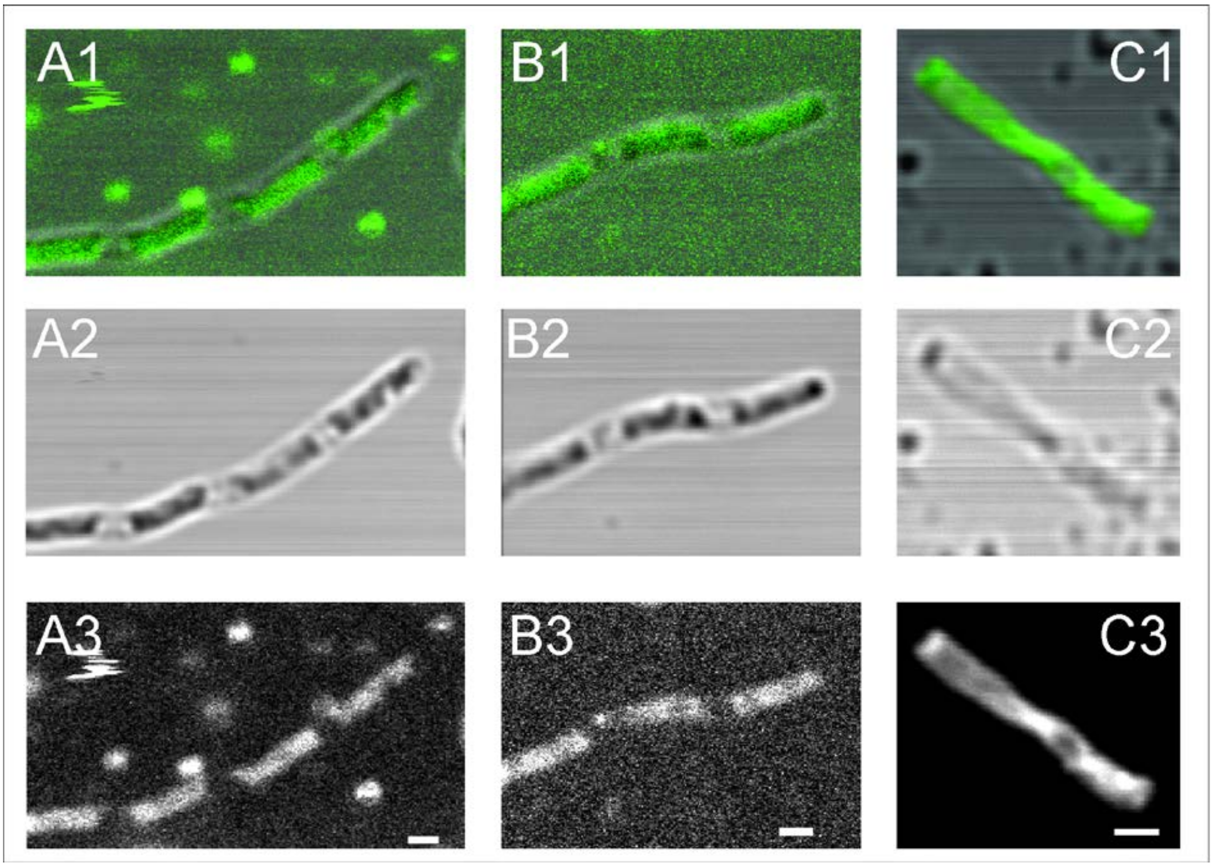

Figure S6. BODIPY C10 stains of cephalexin treated *Escherichia coli* cells subjected to osmotic shock.

In order to test whether the inner or outer membrane was stained by BODIPY C10, *E. coli* cells were stained with BODIPY C10 and were osmotically shocked by increasing the medium osmolality with 15% sucrose. In addition, to obtain elongated cells – in which the visual plasmolysis spaces (VPS, indicators of inner membrane staining) are readily visible – the cells were treated with cephalexin (see Materials and methods section). A1, B1 and C1 show overlays of transmittance (A2, B2 and C2) and fluorescence (A3, B3 and C3) images. Scale bars are 1 µm in length. The transmittance images (A2, B2, C2) indicate that cells have undergone osmotic shock. In the fluorescence images (A3, B3, C3) it can be observed that the stain no longer shows a rod like shape characteristic of cells under normal osmotic conditions (compare with Figure S5 and Fig. 1A-D). Instead the membranes have become invaginated. Moreover, VPS can be observed. This indicates that the rotor BODIPY C10 stains the inner (plasma) membranes of the cells.

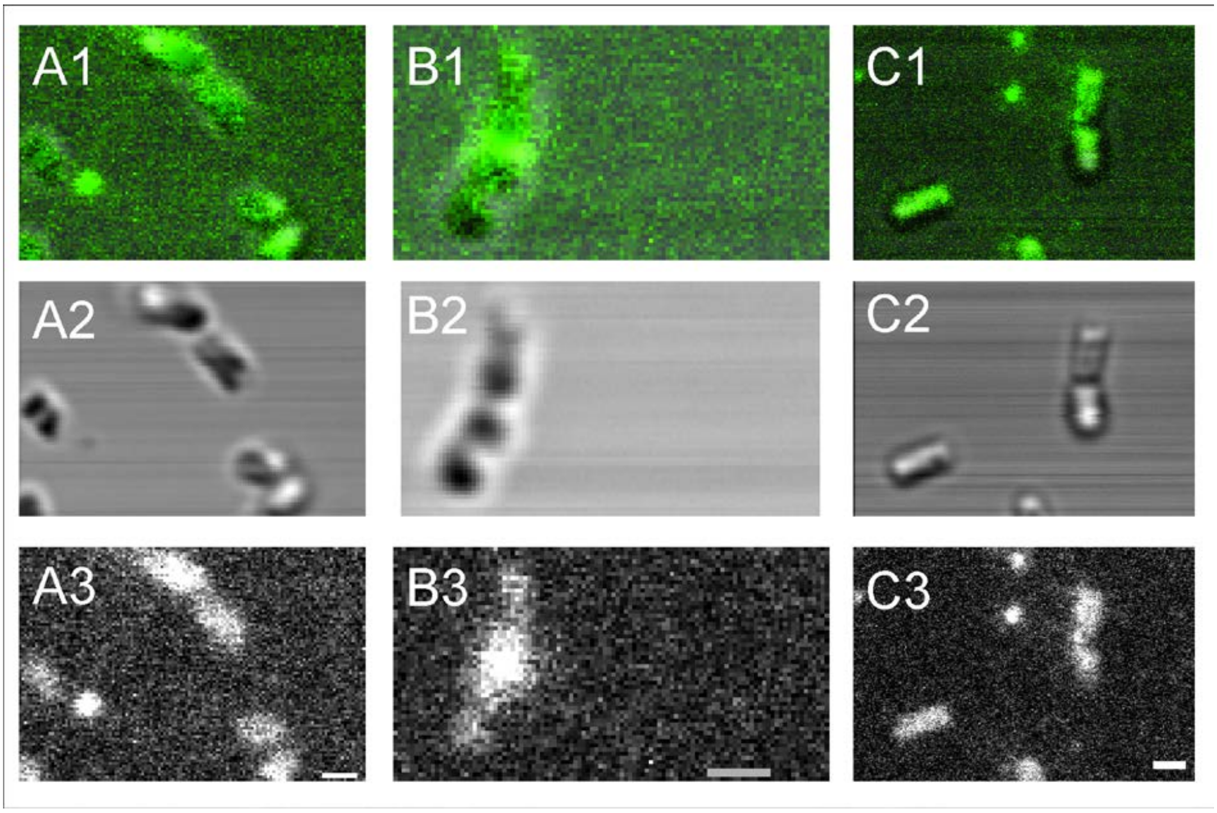

Figure S7. BODIPY C10 stains of healthy *Escherichia coli* cells subjected to osmotic shock.

E. coli cells were stained with the rotor BODIPY C10 and osmotically shocked by increasing medium osmolality with 15% sucrose. A1, B1 and C1 show overlays of transmittance (A2, B2, C2) and fluorescence (A3, B3, C3) images. Scale bars 1 µm. The transmittance images (A2, B2, C2) clearly indicate that cells have undergone osmotic shock. In the fluorescence images (A3, B3, C3) it can be observed that the stain no longer shows a rod like shape characteristic of cells under normal osmotic conditions (compare with Figure S5 and Fig. 1A-D). Instead the membranes appear invaginated and perturbed. This indicates that the rotor BODIPY C10 stains the inner (plasma) membranes of the cells.

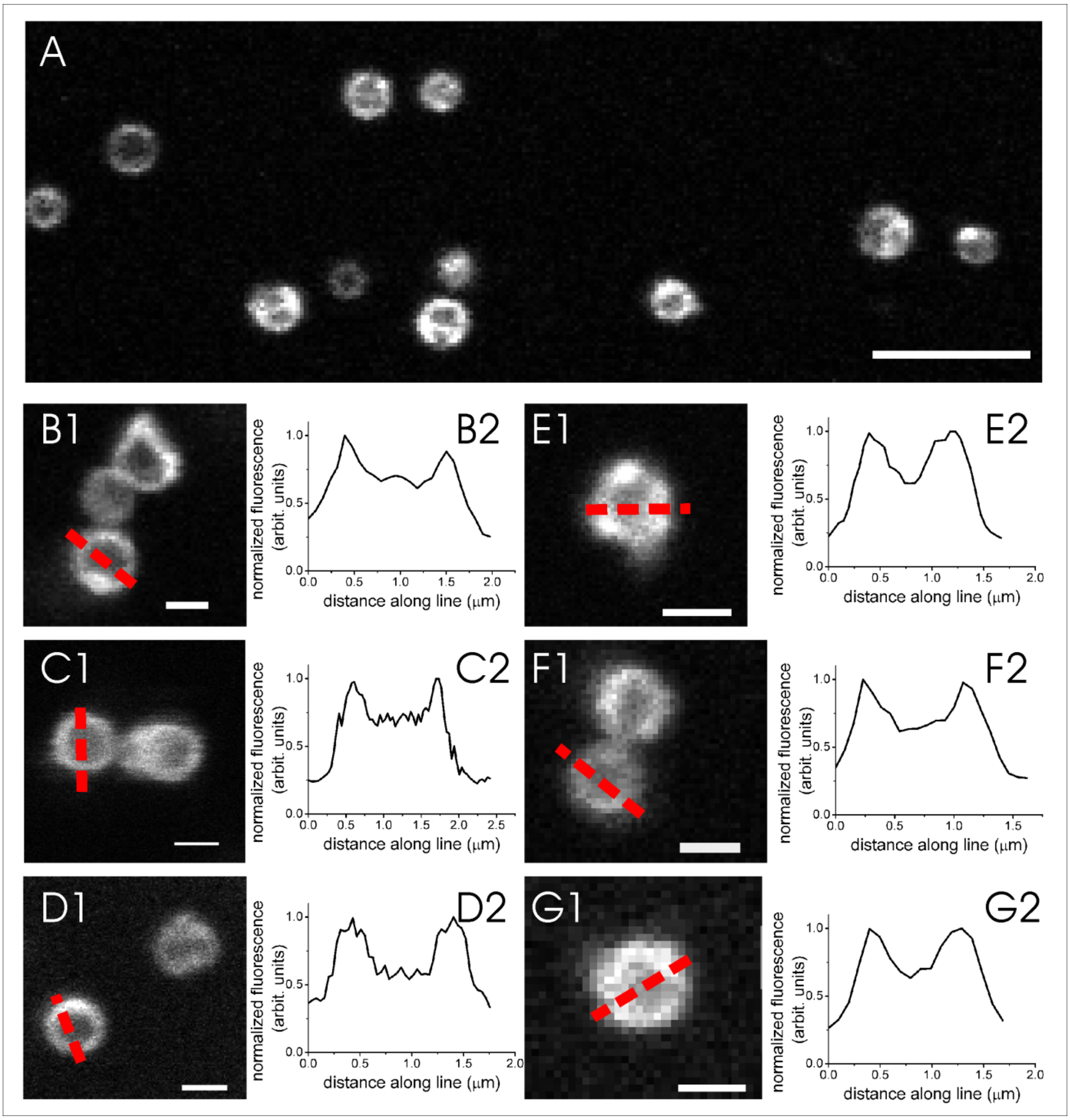

Figure S8. The molecular rotor BODIPY C10 stains membranes in *Escherichia coli* spheroplasts.

Confocal fluorescence images of spheroplasts generated from live *E. coli* cells stained with BODIPY C10. (A) Image showing the membrane stain obtained with BODIPY C10 in several spheroplasts. Scale bar 5 μm. (B-G) Examples of individual spheroplasts stained with BODIPY C10, where a line profile of fluorescence intensity was extracted along the red dotted line. Scale bars 1 μm. The line profiles (subpanels with index 2) have two maxima that correspond to the locations of the membranes in the diffraction limited images. Staining and fluorescence profiles of this sort are indicative of a membrane localised probe.

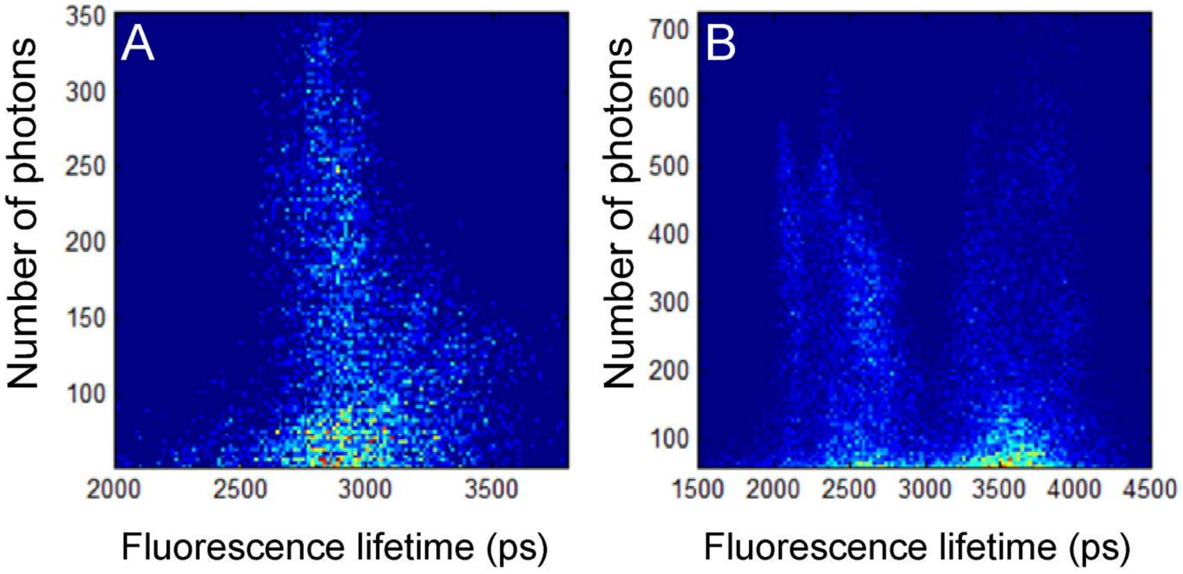

Figure S9. Correlation of fluorescence lifetime and intensity in BODIPY C10 stained live *Escherichia coli* cells.

Correlation of the fluorescence intensity and the fluorescence lifetime for two representative FLIM images (generated using mono-exponential fitting) of *E. coli* stained with BODIPY C10. (A) Cells grown at 37° C and imaged at 23° C; (B) cells grown and imaged at 37° C. The false colour scale represents the number of pixels with a given lifetime and intensity (red – high, blue – low). In both cases the lifetime is observed to be approximately invariant as the intensity increases. This is even the case in (B), where a bimodal lifetime distribution is observed. Thus, fluorescence intensity and lifetime are uncorrelated.
