# Supplementary material for: Measuring the Viscosity of the Escherichia coli Plasma Membrane Using Molecular Rotors
Source: Biophys J. 2016 Oct 4;111(7):1528–40. doi: 10.1016/j.bpj.2016.08.020 (PMC5052448; doi:10.1016/j.bpj.2016.08.020)
Supplement: Document S2. Article plus Supporting Material [file mmc2.pdf]

Biophysical Journal
Article

Biophysical Society

Measuring the Viscosity of the *Escherichia coli* Plasma Membrane Using Molecular Rotors

Jacek T. Mika,[1,*] Alexander J. Thompson,[2] Michael R. Dent,[2] Nicholas J. Brooks,[2] Jan Michiels,[1] Johan Hofkens,[1] and Marina K. Kuimova[2,*]

[1]Centre of Microbial and Plant Genetics (CMPG), KU Leuven, Leuven, Belgium; and [2]Department of Chemistry, Imperial College London, London, United Kingdom

ABSTRACT The viscosity is a highly important parameter within the cell membrane, affecting the diffusion of small molecules and, hence, controlling the rates of intracellular reactions. There is significant interest in the direct, quantitative assessment of membrane viscosity. Here we report the use of fluorescence lifetime imaging microscopy of the molecular rotor BODIPY C10 in the membranes of live *Escherichia coli* bacteria to permit direct quantification of the viscosity. Using this approach, we investigated the viscosity in live *E. coli* cells, spheroplasts, and liposomes made from *E. coli* membrane extracts. For live cells and spheroplasts, the viscosity was measured at both room temperature (23°C) and the *E. coli* growth temperature (37°C), while the membrane extract liposomes were studied over a range of measurement temperatures (5–40°C). At 37°C, we recorded a membrane viscosity in live *E. coli* cells of 950 cP, which is considerably higher than that previously observed in other live cell membranes (*e.g.,* eukaryotic cells, membranes of *Bacillus* vegetative cells). Interestingly, this indicates that *E. coli* cells exhibit a high degree of lipid ordering within their liquid-phase plasma membranes.

INTRODUCTION

The physical environment of live cells is highly heterogeneous, with a large degree of macromolecular crowding, making conditions considerably different from those observed within idealized in vitro systems (1,2). One of the major challenges within quantitative and systems biology is to understand the functioning of a cell as a whole, for example by modeling cellular processes (3,4). However, to achieve this, it is necessary to accurately know the values of the different parameters associated with live cells. In recent years, the methods available for cell biology have become increasingly quantitative, and it is now possible to assign values to the parameters of the key processes in live cells, such as diffusion, catalysis, cell division, replication, transcription, translation, and degradation rates (5).

The viscosities associated with different intracellular compartments are some of the main factors influencing the diffusion and rates of intermolecular reactions within cells. The various biological membranes consisting of phospholipid bilayers are considerably more viscous than the cell cytoplasm and create selective chemical and diffusional barriers for key metabolites within a cell. The exact value of membrane viscosity can change depending on the culturing conditions and can, in turn, influence crucial membrane-associated functions including passive permeability of hydrophobic molecules, active solute transport, and protein-protein interactions (6,7). Perhaps most directly, viscosity influences the translational and rotational diffusion of proteins and lipids in membranes (8).

Living organisms maintain their membranes in a fluid state. *Escherichia coli* bacteria grown at lower temperatures are known to adjust the fatty acid composition of their plasma membranes to maintain membrane fluidity in a process termed "homeoviscous adaptation" (9–11). The Saffman-Delbrück model is a widely accepted model of protein diffusion within membranes (12) and describes the lipid membrane as a thin layer of viscous fluid, surrounded by a less viscous bulk fluid, where the diffusion coefficients of membrane proteins scale logarithmically with the radius they occupy in the membrane and decrease as a function of membrane viscosity. Diffusion coefficients can be readily obtained through the use of microscopy techniques such as fluorescence correlation spectroscopy, fluorescence recovery after photobleaching (FRAP), or single molecule

Submitted June 17, 2016, and accepted for publication August 15, 2016.

*Correspondence: jacek.mika@chem.kuleuven.be or m.kuimova@imperial.ac.uk

Jacek T. Mika and Alexander J. Thompson contributed to this work equally.

Editor: Antoine van Oijen.

http://dx.doi.org/10.1016/j.bpj.2016.08.020

© 2016 Biophysical Society.

This is an open access article under the CC BY license (http://creativecommons.org/licenses/by/4.0/).

tracking (SMT), and a review describing the application of these techniques to the measurement of diffusion in bacteria can be found in an article by Mika and Poolman (13). It follows that to draw quantitative predictions about the size of membrane objects, such as proteins or multiprotein complexes, based on their diffusion coefficient or vice versa, we need to know the value of the membrane viscosity.

Viscosity measurements of total lipid and membrane extracts of *E. coli* were performed using electron paramagnetic resonance (EPR) spectroscopy (10) and relative changes in viscosity were measured using fluorescence anisotropy of diphenylhexatriene (14–16). However, to the best of our knowledge, no direct measurements of viscosity have been performed within *E. coli* membranes in vivo. Recently, Loison et al. (17,18) measured the viscosity of the membranes in the Gram positive bacterium *Bacillus subtilis*, both in spores and in vegetative cells with fluorescence lifetime imaging of molecular rotors. They concluded that the lipid membranes of vegetative cells display a viscosity of ~400 cP, higher than viscosities determined in synthetic lipid bilayers in the liquid-disordered (L_d) phase (19) or within eukaryotic plasma membranes cells (20), suggesting that the plasma membranes of the vegetative cells may exist in a more ordered environment.

Fluorescence lifetime imaging microscopy (FLIM) using molecular rotors is an emerging technique that allows the measurement of viscosity in lipid bilayers, including those in live cells, at a single organelle level with diffraction-limited spatial resolution (17,21,22). The term "molecular rotor" refers to small synthetic fluorophores in which the fluorescence emission is dependent on the viscosity of the surrounding environment. After absorption of a photon, a molecular rotor can undergo either radiative decay via emission of a fluorescence photon or nonradiative decay via intramolecular rotation. The rate of this nonradiative decay is directly affected by the viscosity of the surrounding environment, and the competition that arises between radiative and nonradiative decay leads to the viscosity-sensitive fluorescence properties of molecular rotors. Consequently, both the quantum yield and the fluorescence lifetime of molecular rotors depend on viscosity (22). While the fluorescence quantum yield provides a useful parameter for viscosity calibration in bulk homogeneous samples, it is not suitable for imaging applications, due to the fact that in heterogeneous samples the concentration of a fluorophore is unknown. Fluorescence lifetime determination, on the other hand, is extremely useful for imaging experiments as it can provide a concentration-independent measurement of viscosity on micron scales. Indeed, this approach has previously been applied to viscosity studies in lipid bilayers and monolayers, as well as in membranes of both prokaryotes and eukaryotes (17,20).

In this study we have used the molecular rotor *meso*-(*p*-alkyl-phenyl)-4,4′-difluoro-4-bora-3a,4adiaza-s-indacene (BODIPY C10) (21) to perform FLIM of *E. coli* membranes, which has allowed us to determine the viscosity of the inner membranes on the single cell level. We have analyzed viscosity within individual, live cells and in the population, and have measured the viscosity of spheroplasts, in which the outer membrane was removed by lysozyme treatment. These measurements were made at both room temperature and the *E. coli* growth temperature (37°C). We have also probed the viscosity of lipid vesicles (liposomes) composed of *E. coli* membrane extracts at a range of temperatures, both at and below their growth temperature, providing insights into possible phase transitions within the membrane of *E. coli*. We compare the viscosity values obtained for live *E. coli* cells, spheroplasts, and *E. coli* membrane lipid extracts obtained in this study, and contrast these with membrane viscosities in a variety of other environments such as synthetic membranes, eukaryotic cells, and other bacterial species. We conclude that the plasma membrane of *E. coli* displays a high viscosity, higher than that previously observed in other biological membranes (17,20).

MATERIALS AND METHODS

E. coli growth and labeling

The *E. coli* strain MG1655 (F^-, λ^-, *rph-1*) was grown in Luria Broth medium (10 g/L Bacto Tryptone (Becton Dickinson, Franklin Lakes, NJ), 5 g/L yeast extract (Becton Dickinson), and 10 g/L NaCl (Merck, Kenilworth, NJ)) at 37°C with vigorous shaking (200 rpm). A dense overnight culture was grown from single colonies and on the day of the experiment this was diluted down to low cell density (1:100 dilution) and maintained in early log-phase ($OD_{600} = 0.3$–0.4) by supplementing with fresh medium. Cells were labeled in culture with BODIPY C10 (stock solution in DMSO) for 2 h at a final concentration of 0.2 μM (live cells) or 0.008 μM (spheroplasts) unless stated otherwise. The final concentration of DMSO in the culture did not exceed 0.25%. BODIPY C10 staining did not affect *E. coli* growth at the concentrations used, as confirmed by control experiments (Fig. S1 in the Supporting Material).

Preparation of sample for microscopy

To decrease background from the fluorescent dye and medium, 1 mL of labeled cell culture was harvested by short centrifugation (1.5 min at 8000*g*) and resuspended in nonfluorescent NaPGCl buffer (NaPGCl = 95 mM sodium phosphate, pH 7.0, 50 mM glucose plus 125 mM sodium chloride), which is isosmotic to the Luria Broth growth medium (23). The cells were loaded into 8-well microscopy chambers (Lab-Tek Chambered No. 1.0 Borosilicate Cover Glass System 8 Chamber; Thermo Fischer Scientific, Waltham, MA) and allowed to settle for 5–10 min. Before the start of the experiments, the bases of the microscopy chambers were coated with 0.1% poly-L-Lysine solution (poly-L-lysine; Sigma-Aldrich, St. Louis, MO) to hinder cell motion. Samples were imaged for periods not exceeding 45 min. For measurements performed at 37°C, the chambers and solutions were preequilibrated and then maintained at 37°C using a temperature-controlled microscope stage (E200; Lauda, Lauda-Königshofen, Germany). For room temperature experiments, the temperature-controlled stage was used to maintain the sample at 23°C.

Osmotic shock and cephalexin treatment

For the experiments aimed at revealing the subcellular localization of BODIPY C10 (Fig. 1), cells were grown either as described in *E. coli*

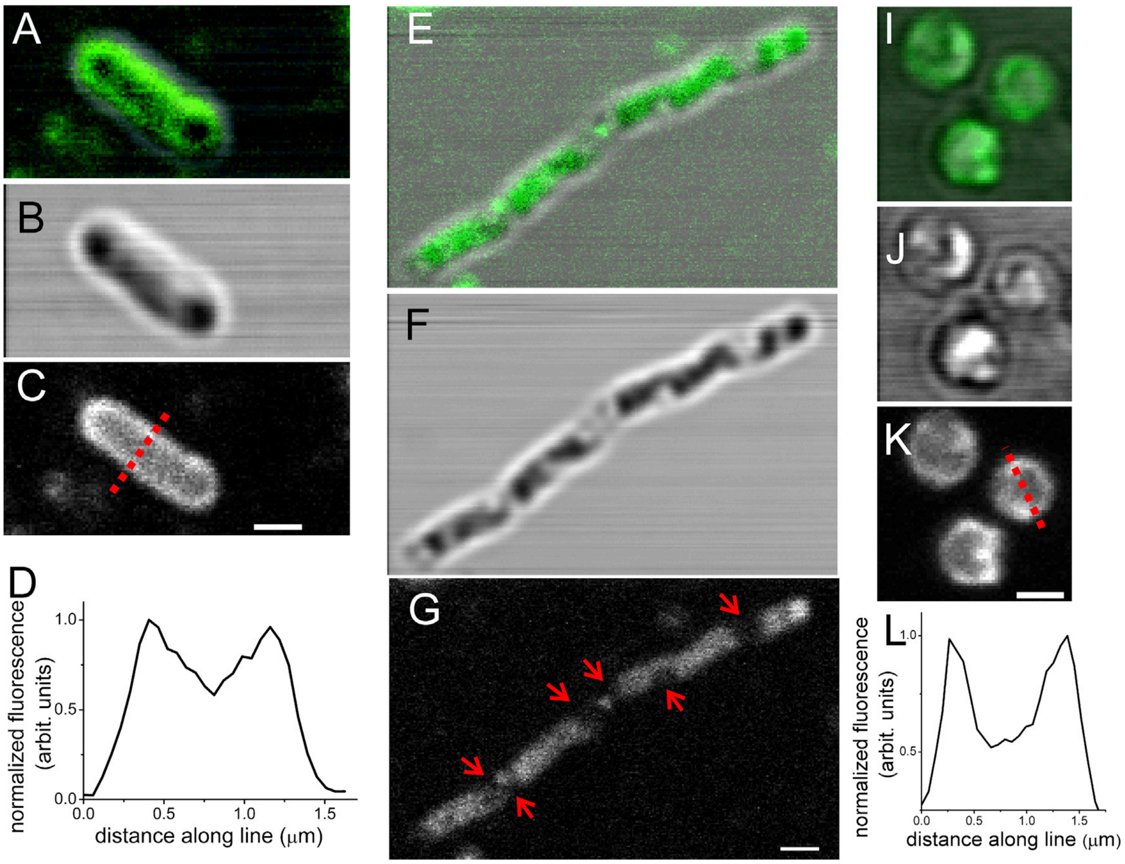

FIGURE 1 (Plasma) membrane localization of the molecular rotor BODIPY C10 in *E. coli*. Confocal fluorescence images of *E. coli* cells and spheroplasts stained with BODIPY C10 indicate (plasma) membrane localization. (*A–D*): a healthy *E. coli* cell stained with BODIPY C10 shows a ringlike stain characteristic of membrane-localized molecules. (*A*) Overlay of transmittance and fluorescence images; (*B*) transmittance image; and (*C*) fluorescence image. (*D*) Fluorescence intensity profile through the red dotted line shown in (*C*): the two fluorescence maxima correspond to the localization of the membrane at either edge of the cell in a diffraction limited image recorded at the midpoint of the cell (from the axial perspective). (*E–G*) An *E. coli* cell treated with cephalexin and subjected to osmotic shock with 15% sucrose. (*E*) Overlay of the transmittance (*F*) and fluorescence (*G*) images. Note that while in the transmittance image (*F*) the outline of the cell still resembles the rodlike shape of a healthy *E. coli* cell, the inner membrane in fact becomes perturbed and invaginated, as observed in the fluorescence image (*G*). The red arrows (*G*) indicate visual plasmolysis spaces, structures characteristic of cells subjected to osmotic upshock, where the inner (plasma) membrane separates from the outer membrane. (*I–L*) Spheroplasts generated from *E. coli* cells. The higher intensity of fluorescence at the edges of the spheroplasts indicates membrane staining. (*I*) Overlay of transmittance (*J*) and fluorescence (*K*) images. (*L*) Fluorescence intensity profile along the red dotted line shown in (*K*). Similarly to (*D*), the two fluorescence maxima correspond to the localization of the membrane at either edge of the cell. Scale bars = 1 μm. To see this figure in color, go online.

Growth and Labeling (healthy cells) or in the presence of 30 μg/mL cell division inhibitor cephalexin for an additional 60–120 min (producing elongated cells) and processed further as described in Preparation of Sample for Microscopy. To obtain osmotically shocked cells, the cells were further resuspended in NaPGCl buffer supplemented with 15% (w/vol) sucrose, in a similar manner to that reported in a previous study from Nenninger et al. (24).

Preparation of spheroplasts

To generate spheroplasts, cells were grown as described in *E. coli* Growth and Labeling with a final concentration of 0.008 μM BODIPY C10 in the growth medium. Spheroplasts were generated by modifying a protocol described in Barthmes et al. (25). One milliliter of cells was harvested by centrifugation at 8000*g* at either 37°C or room temperature for 10 min. The cell wall was digested by adding the following reagents sequentially: 120 μL of 1 M Tris-HCl buffer (pH 7.8), 120 μL of Lysozyme (5 mg/mL), 30 μL of DNase (5 mg/mL), and 120 μL of 0.125 M EDTA (pH 8). The digestion was allowed to proceed for 3 min and then stopped by adding 1 mL of stop solution (20 mM $MgCl_2$, 10 mM Tris-HCL (pH 7.8), 0.7 M sucrose). The final reaction volume was supplemented with BODIPY C10 to a final concentration of 0.008 μM. Spheroplasts were used for microscopy without further washing. Imaging was performed as described in Preparation of Sample for Microscopy.

FLIM measurement

The 8-well microscopy chambers, each containing 200 μL samples, were mounted in a temperature-controlled microscope stage (E200; Lauda) and maintained at the temperature required for the experiment (23 or 37°C). Multiphoton fluorescence lifetime images of individual live *E. coli* cells were then recorded using a time-correlated single photon counting (TCSPC) card (SPC-830; Becker & Hickl, Berlin, Germany). Imaging was achieved using a confocal microscope (TCS SP5 II; Leica Microsystems, Wetzlar, Germany) with an external Titanium:Sapphire laser (Chameleon Vision II; Coherent, Ely, Cambridgeshire, UK), which provided pulsed

excitation permitting two-photon time-resolved fluorescence imaging. The excitation wavelength used was in the range 860–960 nm, which provided efficient two-photon excitation of BODIPY C10. Fluorescence was recorded over the entire emission spectrum of BODIPY C10 (500–650 nm) and an 800-nm short-pass emission filter was used to reject any scattered excitation light.

To ensure that no adverse effects were caused by aggregation of BODIPY C10 in the sample, fluorescence lifetime images were first recorded at a variety of dye concentrations and fit with a monoexponential model (see FLIM Data Analysis). To visualize the lifetime distribution, intensity weighted fluorescence lifetime histograms were plotted for images recorded at a range of concentrations (0.002–0.8 μM). It was observed that the lifetime was constant at concentrations <0.2 μM in live cells and 0.08 μM in spheroplasts (Fig. S2). This indicated that there was no aggregation at or below these concentrations. Thereafter, the dye concentrations used were chosen to provide a sufficient signal/noise (SNR) while still ensuring that no aggregation occurred. Thus, the dye concentrations used for subsequent experiments were 0.2 μM in live cells and 0.008 μM in spheroplasts.

FLIM data analysis

To generate fluorescence lifetime images (Fig. 2), the fluorescence decays recorded in each image pixel were fit to a monoexponential model according to

$$I(t) = I_0 \exp\left(-t/_{\tau}\right), \qquad (1)$$

where I is fluorescence intensity, t is time, I_0 is the fluorescence intensity immediately after excitation, and τ is the fluorescence lifetime. Fitting was achieved using the FLIMfit software tool developed at Imperial College London (26) and fluorescence lifetime images were displayed using a false color scale (see Fig. 2). The goodness-of-fit parameter χ^2 (mean χ^2) was between 1.21 and 1.66 depending on the dataset.

To investigate the lifetime (and viscosity) trends in more detail, we undertook a more detailed biexponential analysis of the recorded fluorescence decays. Regions of interest (ROIs) were manually drawn around individual cells and the decays from all pixels within the ROIs were binned into single decays with high SNR. The resulting decays were then fitted using a biexponential decay model according to

$$I(t) = \alpha_1 \exp\left(-t/_{\tau_1}\right) + \alpha_2 \exp\left(-t/_{\tau_2}\right). \qquad (2)$$

In this equation, α_1, α_2, τ_1, and τ_2 are the amplitudes and lifetimes of the two exponentially decaying components. Good fits were obtained for the decays recorded in all cells and an example fitted decay is shown in Fig. 2 *E*. This procedure yielded single values of all fitted parameters (i.e., α_1, α_2, τ_1, and τ_2) for each individual cell and we also calculated the intensity weighted mean fluorescence lifetime for each cell as

$$\tau_{mean} = \frac{\alpha_1 \tau_1^2 + \alpha_2 \tau_2^2}{\alpha_1 \tau_1 + \alpha_2 \tau_2}. \qquad (3)$$

We investigated trends in both lifetime components (τ_1 and τ_2) as well as in the intensity weighted mean lifetime for a variety of different conditions—i.e., live cells versus spheroplasts, 23 vs. 37°C—and these are displayed as box plots (Figs. 3 and S3).

It has been previously reported that BODIPY C10 has two preferential orientations when located in membranes (27) as explained in more detail in FLIM Measurement of the *E. coli* Plasma Membranes. The long lifetime (high viscosity) component of the measured decays (τ_1) provided the best representation of the membrane viscosity and was used to calculate the

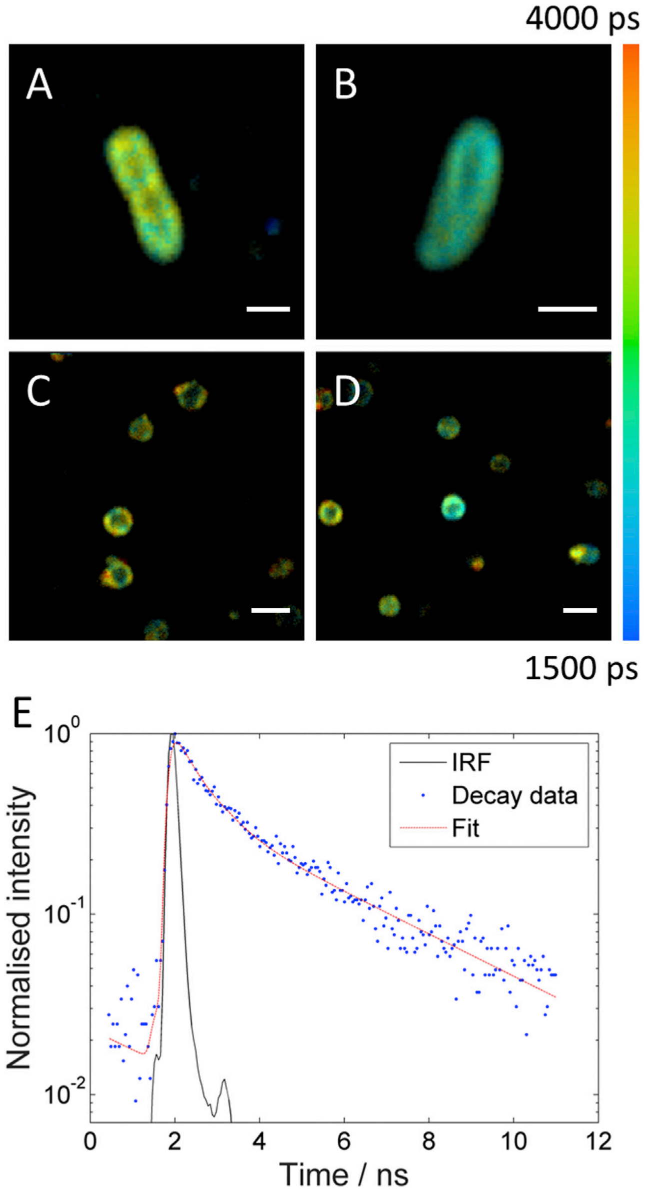

FIGURE 2 Fluorescence lifetime imaging of BODIPY C10 in live *E. coli* cells and spheroplasts. FLIM of BODIPY C10 in live cells (*A* and *B*) and spheroplasts (*C* and *D*). BODIPY C10 preferentially locates to the membrane in both cases. (*A* and *B*) Example fluorescence lifetime images of live *E. coli* cells: (*A*) cell grown at 37°C and imaged at room temperature (23°C); (*B*) cell grown and imaged at 37°C. (*C* and *D*) Two representative fluorescence lifetime images of spheroplasts grown at 37°C and imaged at room temperature (23°C). False color scale represents lifetime in picoseconds. Scale bars are 1 μm in length in (*A*) and (*B*) and 3 μm in length in (*C*) and (*D*). (*E*) Fitted fluorescence decay recorded in a live *E. coli* cell grown at 37°C and imaged at room temperature (23°C). IRF, decay data, and biexponential fit are shown (see key). Decay data was generated by binning all pixels within a single cell in a chosen image to produce a single decay with a SNR high enough to allow biexponential fitting (as described in the Materials and Methods). Normalized data is shown to account for differences in intensity between the fluorescence data and the IRF. A biexponential decay is clearly observed and the long lifetime component of the fit (τ_1) was used to calculate the membrane viscosity. The fitted parameters for the data shown are: $\alpha_1 = 0.31$; $\alpha_2 = 0.69$; $\tau_1 = 3710$ ps; and $\tau_2 = 590$ ps. To see this figure in color, go online.

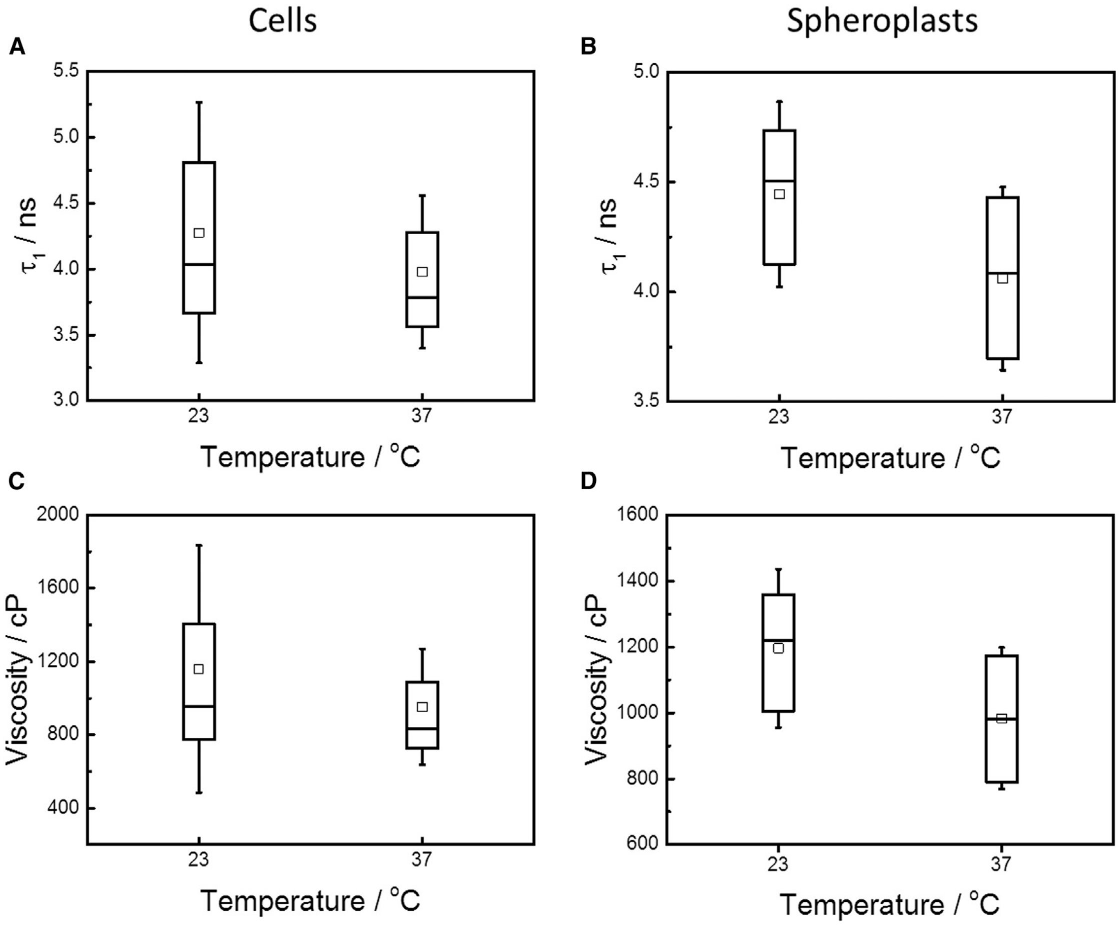

FIGURE 3 Viscosity of the (plasma) membrane of live *E. coli* cells and spheroplasts. Box plots showing the results of fluorescence lifetime measurements of membranes of live *E. coli* cells and spheroplasts stained with BODIPY C10, carried out at room temperature and growth temperature (23 and 37°C, respectively). (*A* and *B*) τ_1; (*C* and *D*) calculated viscosities for *E. coli* cells (*left*; *A* and *C*) and spheroplasts (*right*, *B* and *D*). (*Boxes*) Interquartile range; (*error bars*, i.e., whiskers) 1 SD from the mean; (*horizontal lines*) median values; and (*open squares*) mean values. Number of measurements: cells at 23°C, 21 cells from two independent experiments; cells at 37°C, 57 cells from four independent experiments; spheroplasts at 23°C, 28 spheroplasts from two independent experiments; and spheroplasts at 37°C, 58 spheroplasts from four independent experiments.

viscosity of the *E. coli* cell membrane. Viscosities were calculated according to the previously reported viscosity-lifetime calibration equation—which was obtained by measuring fluorescence decays from BODIPY C10 in methanol/glycerol mixtures of known viscosity (28)—with τ_1 used as the lifetime value:

$$\log \eta = \frac{\log \tau_1 + 0.75614}{0.4569}. \quad (4)$$

In Eq. 4, η represents the viscosity in cP and τ_1 represents the long lifetime component in ns. The membrane viscosities observed under different conditions (i.e., live cells versus spheroplasts, 23 vs. 37°C) are displayed in Fig. 3, Table 1, and Table S1 in the Supporting Material.

To achieve accurate fitting of the fluorescence decays, it is necessary to deconvolve the measured data from the instrument response function (IRF). For both mono- and biexponential fitting, the IRF was estimated by recording the instantaneous second harmonic generation signal from a sample of urea crystals. This was achieved using an excitation wavelength matched to that used in the *E. coli* experiments (860–960 nm). The detection wavelength was set at half the excitation wavelength with a detection bandwidth of 20 nm (i.e., for 860 nm excitation, a detection wavelength range of 420–440 nm was used).

Viscosity measurement in liposomes composed of *E. coli* lipid extracts

All liposome studies used a BODIPY C10 rotor concentration of 0.5 mol % (1:200 rotor/lipid) to prevent dye aggregation (19,29) and to avoid significant disruption of the bilayer structure. Large unilamellar vesicles were formed using the gas extrusion method (30). A solution of *E. coli* total lipid extracts (Avanti Polar Lipids, Alabaster, AL) and BODIPY C10 was prepared in chloroform, which was then evaporated off under nitrogen. Multilamellar vesicles were then prepared by hydrating the lipid film using enough water to give a 1 mM solution of lipid and vortexing for 1 min above the gel transition temperature of the lipid. This was then extruded 10 times through a polycarbonate membrane with a pore diameter of 200 nm using a LIPEX extruder (Northern Lipids/Transferra Nanosciences, Burnaby, British Columbia, Canada), ensuring it was above the gel transition temperature of the lipid.

Lifetime measurements were obtained with an IBH 5000 F TCSPC device (Horiba Jobin Yvon, Edison, NJ), using a pulsed NanoLED source at 404 nm for excitation. All TCSPC measurements gave 10,000 counts in the peak channel, and Horiba DAS6 software (Horiba Jobin Yvon) was used to fit the decays using a biexponential decay model (see Eq. 2). Viscosities were calculated as described for live cells

TABLE 1 BODIPY C10 Fluorescence Lifetimes and Corresponding Viscosities in Membranes of Different Systems Recorded at 23 and 37°C

Lipid System	BODIPY C10 Lifetime (ns)	Viscosity (cP)	Reference
At 23°C			
DOPC	1.67	140	(19)
SPM	5.27	1720	(19)
SPM/20% Chol	4.85	1430	(19)
SK-OV cells	2.2	250	(20)
Bacillus spores inner membrane	4.0	960	(17)
Bacillus vegetative cell membrane	2.7	400	(17)
E. coli cells	4.28	1160	This study
E. coli spheroplasts	4.44	1200	This study
E. coli membrane extracts	2.46	320	This study
At 37°C			
DOPC	1.11	60	(19)
SPM	3.33	630	(19)
SPM/20% Chol	3.26	600	(19)
E. coli cells	3.98	950	This study
E.coli spheroplasts	4.06	980	This study
E. coli membrane extracts	1.98	200	This study

BODIPY C10 fluorescence lifetimes and corresponding viscosities observed in a variety of membrane systems. This includes the data from live *E. coli* cells, spheroplasts, and membrane extract liposomes recorded in this study as well as data from other lipid vesicles (liposomes) and both eukaryotic and prokaryotic cells reported in the literature (17,19,20).

(Eq. 4) using the methanol/glycerol calibration described in Hosny et al. (28) and with τ_1 taken to represent the viscosity, in agreement with previous work in ordered model membrane systems (27). The *E. coli* total lipid extracts were obtained from bacteria grown at 37°C and lifetime measurements were carried out in bulk solutions over a range of temperatures (5–40°C). The measured lifetimes (τ_1, τ_2, and τ_{mean}) and the viscosity calculated from τ_1 were plotted as functions of temperature (Figs. 4 and S4).

Calculation of diffusion coefficients

To compare our measured membrane viscosity values to previous studies, we used the Saffman-Delbrück diffusion equation (12) to convert the viscosity values reported here into diffusion coefficients. Using this approach, the diffusion coefficient, D_{sd}, is related to the membrane viscosity, η_m, according to

$$D_{sd} = \frac{k_B T}{4\pi \eta_m h}\left[\ln\left(\frac{2L_{sd}}{a}\right) - \gamma\right], \quad (5)$$

where h represents membrane thickness, k_B is Boltzmann's constant, T is temperature, a is the radius of the diffusing particle, $\gamma \approx 0.577$ is the Euler-Mascheroni constant, and L_{sd} is the Saffman-Delbrück diffusion length, which is defined in terms of the membrane viscosity, the membrane thickness, and the viscosity of the surrounding fluid (η_f) as

$$L_{sd} = \frac{h\eta_m}{2\eta_f}. \quad (6)$$

To calculate a diffusion coefficient, the membrane viscosity was first calculated from the measured BODIPY C10 fluorescence lifetime, and the following values for particle radius (a), fluid viscosity (η_f) and

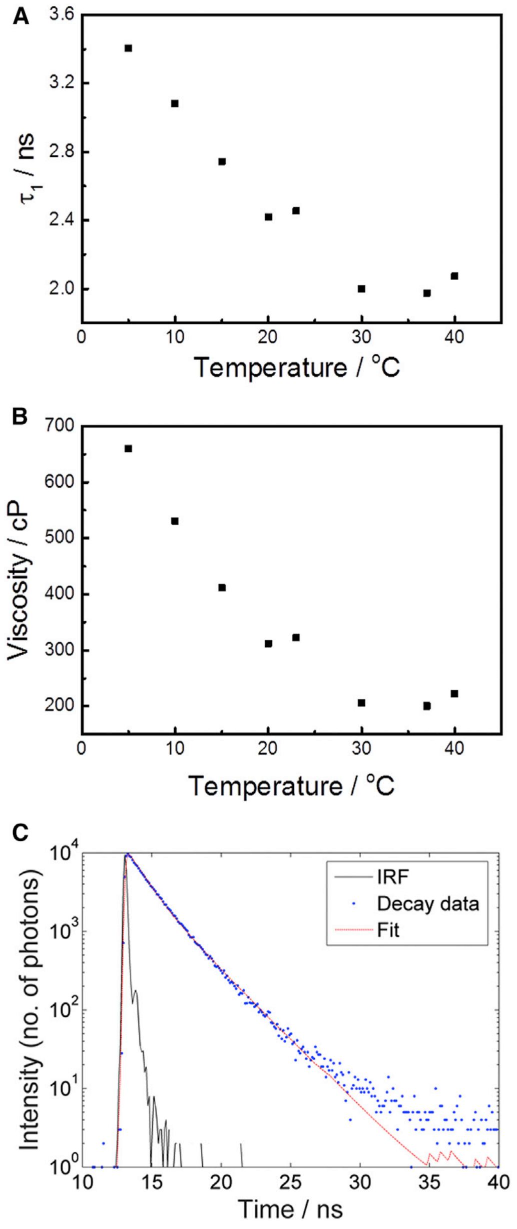

FIGURE 4 Viscosity of liposomes made of *E. coli* lipid extracts as a function of temperature. TCSPC measurement of BODIPY C10 labeled liposomes composed of *E. coli* membrane lipid extracts, measured as a function of temperature. Graphs show (*A*) the fluorescence lifetimes (τ_1) of the vesicles (liposomes) and (*B*) the corresponding viscosities with respect to temperature. Liposomes were prepared using lipid extracts from *E. coli* cells grown at 37°C. (*C*) Fitted fluorescence decay recorded in a sample of liposomes at room temperature (23°C). The IRF, decay data, and biexponential fit are shown (see key). Data was collected using an IBH 5000 F TCSPC device (Horiba Jobin Yvon) with the acquisition time set to provide a decay (and IRF) with 10^4 photons in the peak. As for the live cell data, a biexponential decay is observed and the long lifetime component of the fit (τ_1) was used to calculate the membrane viscosity. The fitted parameters for the data shown are: $\alpha_1 = 0.41$; $\alpha_2 = 0.59$; $\tau_1 = 2460$ ps; and $\tau_2 = 1070$ ps. To see this figure in color, go online.

membrane thickness (h) were then used for each of the membrane systems investigated (31):

$$\begin{aligned} a &= 0.62\ \text{nm}; \\ \eta_f &= 0.8903\ \text{cP}; \\ h(E.\ coli) &= 3.75\ \text{nm}; \\ h(\text{Extracts}) &= 3.35\ \text{nm}. \end{aligned}$$

Note that the *E. coli* membrane thickness was assumed constant for both cells and spheroplasts at 3.75 nm (31). The calculated diffusions coefficients are shown in Table 2 alongside values reported previously using BODIPY FL-C_{12} (24,32), which has a similar structure to BODIPY C10.

RESULTS

BODIPY C10 labels *E. coli* plasma membranes

BODIPY C10 is a well-studied molecular rotor, whose fluorescence lifetime depends on the viscosity of the environment in the range 10–1000 cP (28). BODIPY C10 is known to partition well into lipid bilayers in model membranes and in bacterial cells (17,19), and in this study we used BODIPY C10 to probe the viscosity of *E. coli* membranes. Confocal fluorescence microscopy of *E. coli* stained with this probe (Fig. 1) reveals a ringlike structure bordering the rodlike shape of each bacterial cell. Similar staining was observed previously for the commercially available, membrane-localized probe BODIPY FL-C_{12} (24) and for the *E. coli* bona fide inner membrane protein LacY tagged with GFP (23). Fluorescence intensity profiles extracted along lines drawn through the middle of the cells (e.g., the red line in Fig. 1 *C*) show two maxima (Figs. 1 *D* and S5) that correspond to the position of the membrane.

Because *E. coli* has two membranes—the outer membrane (the cell wall) and the inner membrane (the plasma membrane)—we sought to investigate which of the two structures is labeled by BODIPY C10. As a first experiment, we followed the approach of Nenninger et al. (24). In their study, the probe BODIPY FL-C_{12}, which has a very similar structure to BODIPY C10, was shown to localize to the plasma membranes of *E. coli* based on an osmotic upshift experiment. When *E. coli* cells are suddenly placed in a medium of much higher osmolality than their growth medium, water leaves the cytoplasm, the cytoplasm volume decreases and eventually (if the osmotic shock applied is high enough), the cells plasmolyze (33). As the cytoplasm decreases in volume, it loses its characteristic rodlike shape, its appearance becomes perturbed and eventually visual plasmolysis spaces can be observed (23,34). While the inner membrane follows the cytoplasm, loses the rodlike shape and appears perturbed (23), staining of the outer membrane of osmotically shocked *E. coli* with FM4-64 revealed that the outer membrane does not alter its volume and shape as significantly and retains the characteristic rodlike shape (35).

To facilitate the observation of the subcellular localization of the probe with diffraction-limited microscopy, *E. coli* cells stained with BODIPY C10 were treated with cephalexin (an antibiotic that disturbs cell growth resulting in elongated cells) and subsequently subjected to osmotic shock with a medium supplemented with 15% sucrose. In these elongated, osmotically shocked cells, the membrane stain is no longer rodlike, the membrane appears perturbed (Figs. 1, *E–G*, and S6), and visual plasmolysis spaces are readily present (*red arrows* in Figs. 1 *G* and S6). In addition, a similar reaction was also observed for healthy (noncephalexin-treated) *E. coli* cells (Fig. S7). This implies that BODIPY C10, in a similar manner to BODIPY FL-C_{12}, localizes to the inner membranes of *E. coli*.

In this study, alongside measurements in live *E. coli* cells, we have also probed spheroplasts generated from those live cells by digestion with lysozyme (25). This digestion removed the outer membranes of *E. coli* (see Materials and Methods for details), yielding spheroplasts (Figs. 1, *I–L*, and S8), the membranes of which consist of the plasma (inner) membrane of the original bacteria. It can be observed that BODIPY C10 also exhibits a membrane stain in spheroplasts (a clear circular staining around each spheroplast),

TABLE 2 Comparison of the Diffusion Coefficients Observed in *E. coli* Cells, Spheroplasts, and Lipid Extract Liposomes Using the Two Structurally Similar Probes BODIPY C10 and BODIPY FL-C_{12}

Lipid System	Probe	Technique	Diffusion Coefficient ($\mu m^2/s$)	Reference
At 23°C				
E. coli cells	BODIPY C10	FLIM	0.63	This study
E. coli spheroplasts	BODIPY C10	FLIM	0.61	This study
E. coli extracts	BODIPY C10	TCSPC	2.1	This study
E. coli cells	BODIPY FL-C_{12}	SMT	1.5	(32)
E. coli cells	BODIPY FL-C_{12}	FRAP	0.6	(24)
At 37°C				
E. coli cells	BODIPY C10	FLIM	0.78	This study
E. coli spheroplasts	BODIPY C10	FLIM	0.76	This study
E. coli extracts	BODIPY C10	TCSPC	3.0	This study
E. coli cells	BODIPY FL-C_{12}	FRAP	1.2	(24)

Diffusion coefficients observed in *E. coli* cells, spheroplasts, and lipid extract liposomes using both BODIPY C10 and BODIPY FL-C_{12}. The BODIPY C10 data is taken from this study and was calculated using the Saffman-Delbrück diffusion equation to convert measured viscosities into diffusion coefficients (see Materials and Methods). BODIPY FL-C_{12} data was reported previously in the references indicated in the table.

as seen from both images and fluorescence intensity profiles (Figs. 1, *I–L*, and S8).

In a second, complementary approach to establish the localization of BODIPY C10, we determined the fluorescence lifetime of BODIPY C10 in the membranes of live cells and compared this to the lifetime measured within spheroplasted cells, which we describe in more detail. Briefly, the fluorescence lifetimes obtained for both live cells and spheroplasts were very similar (Figs. 2 and 3), implying that BODIPY C10 is located in comparable environments in both cases. Thus, this provided further evidence that BODIPY C10 resides in the plasma membrane of *E. coli* cells.

Taken together, the staining and osmotic shock experiments (Fig. 1), the FLIM measurements in the spheroplasts as compared to live cells (Figs. 2 and 3), and the fact that a chemically very similar probe (BODIPY FL-C_{12}) was also shown to reside in the plasma membrane of *E. coli* (24), all provide strong indications that BODIPY C10 labels the inner (plasma) membrane of *E. coli*.

FLIM measurement of the *E. coli* plasma membrane

We performed FLIM measurements on live *E. coli* cells and spheroplasts stained with BODIPY C10 to determine the viscosity of the plasma membranes. In these measurements the fluorescence lifetimes recorded in each pixel of the image of interest can be compared with lifetimes on the viscosity calibration curve of BODIPY C10, obtained by determining the BODIPY C10 lifetime within methanol/glycerol solutions of known viscosity (28) (see FLIM Data Analysis).

We first confirmed that, at the low incubation concentrations of BODIPY C10 used for staining, aggregation of the dye did not play any role and the FLIM data obtained were reliable. While using higher concentrations of BODIPY C10 produces brighter images, above a certain concentration threshold aggregation of dye molecules can occur. Aggregation produces an additional fluorescence emission peak at 630–670 nm, which acts to quench the monomer emission centered at ~515 nm. This causes a reduction in monomer lifetime and renders the viscosity-lifetime calibration data unreliable (19). Thus, it was vital to establish that the concentration of BODIPY C10 used was low enough to ensure that aggregation had no effect on the measurements. To this end, we recorded FLIM images of healthy cells and spheroplasts at a variety of BODIPY C10 concentrations. We observed that the recorded lifetimes increased with decreasing concentration (implying a reduction in aggregation) only up to a point. For both healthy cells and spheroplasts, the lifetime became constant below a certain concentration threshold, indicating that no aggregation-induced quenching was occurring (Fig. S2). Optimal BODIPY C10 concentrations were then chosen for both healthy cells and spheroplasts that provided sufficient signal while also ensuring that no aggregation took place. These were established to be 0.2 μM for live, nonspheroplasted cells and 0.008 μM for spheroplasts. Hence, all the subsequent data presented herein were recorded at these concentrations. Finally, we also confirmed that at the concentration used for the FLIM measurements ($\leq$0.2 μM), BODIPY C10 had no adverse effects on cell growth (Fig. S1).

Having determined the optimum BODIPY C10 concentration to be used for imaging, we recorded multiphoton FLIM images of live *E. coli* cells and spheroplasts both at room temperature (23°C) and at 37°C (with cells grown at 37°C in both cases). It was previously established that BODIPY C10 measures viscosity in a temperature-independent manner, i.e., its fluorescence decay is affected by viscosity only and not by temperature (36). Representative FLIM images (generated using pixelwise monoexponential fitting) are shown in Fig. 2, *A–D*, and a number of preliminary observations can be made. First, the probe preferentially labels the edges of the structures indicating membrane localization as mentioned above. In addition, the lifetimes in individual cells (both healthy cells and spheroplasts) appear uniform with all areas of the membrane exhibiting broadly similar lifetimes. Interestingly, as discussed above, the lifetimes recorded in healthy cells and spheroplasts are in qualitative agreement, suggesting that the same region of the cells (the inner membrane) is stained in both cases.

To investigate these lifetime trends further, we undertook a more detailed analysis of the fluorescence decays recorded in a population of cells using biexponential fitting (see FLIM Data Analysis for details). Briefly, ROIs were manually drawn around individual cells and all photons within the ROIs were binned into single decays. Those decays were then fitted to a biexponential decay model yielding two fitted lifetime values for each cell (τ_1 and τ_2). An example fitted decay is shown in Fig. 2 *E*. It has been reported previously that BODIPY C10 has two preferential orientations when located within more ordered lipid bilayers or membranes (27). In one orientation, the viscosity-sensitive region of the BODIPY molecule is situated within the tail region of the membrane, while in the second orientation the viscosity sensitive moiety is parallel to the membrane core, which is typically a lower viscosity environment. Thus, with a high enough SNR—which we obtained by binning all pixels in individual cells—we observed biexponential fluorescence decays from BODIPY C10 in *E. coli* membranes, which is a hallmark of a more ordered environment. Further, the long lifetime component of these decays (in this case, τ_1) will provide the best representation of the viscosity of the membrane, i.e., the lipid tail region of the bilayer. Thus, this analysis permitted the calculation of membrane viscosities in individual cells as well as the comparison of viscosities across different populations (e.g., live cells versus spheroplasts, imaging at 23°C versus imaging at 37°C). The results of this procedure applied to a population of

both cells and spheroplasts are discussed in detail in the following section, and we note that due to an insufficient photon count we were not able to perform FLIM imaging combined with biexponential decay analysis.

Viscosity of the *E. coli* plasma membrane

As discussed above, we recorded FLIM images of BODIPY C10 in live cells grown at 37°C and spheroplasts generated from them, both at the growth temperature—where the plasma membrane would be expected to be in a fluidlike state—and at room temperature (23°C), to investigate the membrane phase properties. The fluorescence decays recorded for individual cells and spheroplasts were fitted as explained in other sections (see above and Materials and Methods), and Fig. 3 shows the average lifetimes and viscosities recorded in a population. There was a large spread in the measured lifetimes (and, hence, in the viscosities) between individual cells. The mean value of the long lifetime component (τ_1, the component that provides the best representation of membrane viscosity) in live cells measured at growth temperature was 3980 $\pm$ 580 ps ($n = 57$ cells from four independent measurements), which corresponds to a mean viscosity of 950 $\pm$ 320 cP. For spheroplasts under similar conditions, the mean value of the long BODIPY C10 lifetime component (τ_1) was 4060 $\pm$ 420 ps ($n = 58$ spheroplasts from four different measurements) corresponding to a mean viscosity of 980 $\pm$ 210 cP.

For cells measured at room temperature the mean value of τ_1 was found to be 4280 $\pm$ 990 ps ($n = 21$ cells from two independent experiments), which corresponds to a mean viscosity of 1160 $\pm$ 670 cP. For spheroplasts at room temperature, the mean value of τ_1 was 4450 $\pm$ 420 ps ($n = 28$ spheroplasts from two independent measurements) yielding a viscosity of 1200 $\pm$ 240 cP. The values here were measured in a population of cells. For other statistics describing the measurement (e.g., median and interquartile range), see Table S1. As discussed briefly above, the viscosities measured in the spheroplasts match those measured in live cells. Because it is likely that the outer membrane has a very different viscosity value from that of the inner membrane and because spheroplasts have the outer membranes removed by lysozyme treatment, we interpret the close overlap of the viscosities recorded in live cells and spheroplasts as an indication that BODIPY C10 localizes in the inner membrane of *E. coli*.

The spread of the measured lifetimes and thus viscosities is rather large and this indicates a large cell-to-cell heterogeneity. Additionally, the viscosity measured at growth temperature is lower than for the cells measured at room temperature (Fig. 3 *C*). This difference may indicate that a phase transition has taken place within the membrane, as liquid-ordered and gel phases are known to show a higher viscosity than liquid-disordered phases (27). However, the change is relatively small compared to the spread of the data and a Students' *t*-test performed on the fluorescence lifetime data for healthy cells indicated that the change was not statistically significant ($p = 0.11$). Thus, in isolation this does not provide conclusive evidence of a phase transition. Nonetheless, previous studies investigating the phase transition of *E. coli* membranes reported that the phase transition occurs ~14–16°C below the growth temperature (10,24), which is in qualitative agreement with our tentative observation. Interestingly, the spread of the measurement is higher for cells measured at 23°C than for those measured at 37°C, which could imply that some cells respond more readily to the temperature shift than others. Furthermore, spheroplasts also exhibited a similar increase in viscosity (and lifetime) at the lower measurement temperature and in this case the lifetime shift was found to be statistically significant ($p = 0.00012$). Taken together, these data suggest that a temperature dependent phase transition may indeed take place in *E. coli* plasma membranes and that further investigation of this transition is warranted (for example, through measurements on larger populations of cells and over a wider range of temperatures).

It has also been reported that *E. coli* cells can display a large heterogeneity of physical parameters between isogenic cells in the same culture (37,38). An example of such heterogeneity is the large spread of translational diffusion coefficients of cytosolic proteins observed in bacteria (13,34,39), where diffusion coefficient (D) values of individual sister cells in a culture all stemming from the same mother clone can differ from one another by up to an order of magnitude. Using FLIM, which is well suited to compare viscosities at the single cell level, we tested whether the viscosity of membranes in a population of isogenic live *E. coli* cells is heterogeneous. We observed that the lifetime, τ_1, within a population of cells grown and measured at 37°C showed a spread (2 $\times$ SD) of ~1160 ps (Fig. 3 *A* and Table S1), which corresponds to a spread in viscosity of 630 cP (Fig. 3 *B*; Table S1). In addition, the interquartile range was found to be 720 ps (Fig. 3; Table S1), corresponding to a spread in viscosity of 360 cP (Fig. 3 *B*; Table S1). As discussed above, the spread in viscosity is greater for cells imaged at room temperature than for those interrogated at their growth temperature. The observed spread in viscosity values in live cells is also larger than that measured in spheroplasts (Fig. 3; Table S1). Furthermore, the variances observed in spheroplasts at 23°C and 37°C are approximately equal suggesting that the membranes of spheroplasts may respond to the temperature shift in a more homogeneous manner than those of live cells.

Importantly, the spread (along with the measured viscosities in general) was not dependent on the amount of label taken up by the cells, as there was no correlation between cell fluorescence intensity and the fluorescence lifetime (Fig. S9). Likewise, the spread was consistent in the results for τ_1, τ_2, and the mean-weighted τ, suggesting that it was not uncertainty in τ_2 that caused the spread,

and that our chosen fitting model did not adversely affect our results.

Viscosity of liposomes composed of *E. coli* lipid extracts

The viscosities of *E. coli* lipid extracts and liposome preparations have been measured previously with EPR (10). To compare the viscosity values obtained in this study on live cells with those previous measurements, we have probed the fluorescence lifetime of the BODIPY C10 molecular rotor incorporated into liposomes made of *E. coli* total lipid extracts, by performing bulk measurements using TCSPC, as described in Viscosity Measurement in Liposomes Composed of *E. coli* Lipid Extracts. Fluorescence decays from BODIPY C10 in liposomes composed of *E. coli* lipid extracts were recorded over a range of temperatures and fitted using a biexponential decay model (see above and Materials and Methods) to yield two fluorescence lifetimes (Fig. 4 *C*). As in the case of measurements on live cells and spheroplasts, we have taken τ_1 as a representative indicator of membrane viscosity.

Measurement at 37°C (the growth temperature of bacteria from which the lipids were extracted) gave a viscosity value of 200 cP while measurement at 30°C gave a similar value of 210 cP (Fig. 4). These values correspond well to the measurements performed by Sinensky (10), where the viscosity of lipid extracts made of cells grown at 37°C and measured at 37 ^{o} C was reported to be 180 cP (other values reported in that study were between 180 and 200 cP for lipid extracts measured at the temperature of cell growth). Below 30°C we observe a gradual increase in the measured viscosity with decreasing temperature. For example, at 23°C a viscosity of 320 cP was observed and the viscosity recorded at 5°C (the lowest temperature investigated) was 660 cP (Fig. 4). This increase in membrane viscosity may be an indication of a phase transition in the liposomes composed of *E. coli* lipids. According to literature data the phase transition of *E. coli* membranes occurs ~14–16°C below the growth temperature (10,24), which for cells grown at 37°C should be at ~23°C. Hence, at higher temperatures (i.e., 37°C (growth temperature) and 30°C) the membranes of the liposomes composed of *E. coli* lipid extracts are most likely in the fluid state and exhibit (relatively) low viscosities. At lower temperatures the membranes undergo a phase transition to a more ordered state causing an increase in viscosity. From Fig. 4, we observe that the phase transition occurs in the range 20–30°C, which is in qualitative agreement with previous work (10,24). This observation of a phase transition in liposomes coupled with the changes recorded in live cell viscosities at different temperatures (discussed above) suggest that further investigation of the response of the cell membrane viscosity to temperature is warranted.

Finally, it is also interesting to note that we observed considerably higher viscosities in live cells (and spheroplasts) than in liposomes. This is most likely explained by the facts that the liposomes have a simpler structure than live cell membranes, contain no (or significantly fewer) proteins, and might have a contribution of lipids from the outer membrane. Hence, the liposomes provide a less crowded environment for the molecular rotor and have a lower effective viscosity, and we comment on this observation in more detail in the Discussion.

DISCUSSION

Viscosity is a highly important parameter within the plasma membrane, controlling the rate of diffusion of small molecules and proteins. Despite this, there are still significant challenges associated with directly measuring membrane viscosity within cells, and to the best of our knowledge, this study represents the first attempt to directly quantify viscosity within live *E. coli* plasma membranes. In live *E. coli* cells at 23 and 37°C, we found the mean plasma membrane viscosities to be 1160 and 950 cP, respectively, compared with values in spheroplasts at 23 and 37°C of 1200 and 980 cP, respectively. These values agree well with those calculated from membrane protein diffusion coefficients by Oswald et al. (32), who estimated membrane viscosities in the range 1000-1200 cP using the Saffman-Delbrück diffusion model.

Table 1 shows mean BODIPY C10 fluorescence lifetime values along with the corresponding viscosities reported in a range of membrane systems and a number of observations can be made. First, the viscosities measured in live *E. coli* cells are considerably higher than those observed in model liquid-disordered phase membranes (1,2-dioleoyl-sn-glycero-3-phosphocholine, DOPC), in eukaryotic (SK-OV-3) cells and in *Bacillus* vegetative cells, as measured by FLIM of BODIPY-based fluorescent molecular rotors (17,19,20). Interestingly, the *E. coli* membrane viscosities are closer in value to those measured in the membranes of *Bacillus* spores and model liquid-ordered phase bilayers (SPM/20% cholesterol), suggesting that *E. coli* plasma membranes display a high degree of lipid ordering.

The increased viscosity of the *E. coli* plasma membrane (relative to the eukaryotic cell membrane) may be caused by the high abundance of phosphatidylethanolamine (PE) lipids within the *E. coli* membrane, which make up ~70% of the lipid content (40,41). PE lipids have previously been shown to reduce the diffusion coefficients of lipids within model systems (42,43) and it was speculated this is due to increased viscosity (43). On the molecular level, there is an increased molecular packing (44) and additional hydrogen bonds (42–44) that are associated with the PE lipid headgroup compared to the PC headgroup, which is commonly found in eukaryotic membranes.

Regarding temperature effects, while we recorded a lower cell membrane viscosity at 37°C than at 23°C (both in live cells and in spheroplasts), we also observed considerable

intercell variation meaning that the temperature difference measured in healthy, nonspheroplasted cells was not statistically significant ($p = 0.11$). Nevertheless, the temperature shift effect on viscosity in spheroplasts was found to be statistically significant ($p = 0.00012$) and, in addition, a clear temperature trend was observed in lipid extract liposomes. It was discussed that this may indicate a phase transition (both in liposomes and in live cells), and for this reason further investigation of the behavior of the viscosity of live plasma membranes in response to temperature is certainly warranted.

The spread of the data is also interesting in itself as it implies that there is a large intercell heterogeneity in the viscosities of *E. coli* membranes. This is in agreement with previous work—for example, the literature (13,23,34,37,38)—showing that *E. coli* can display a large heterogeneity in the physical parameters of the cell (e.g., the large cell-to-cell variation of diffusion coefficients of cytoplasmic proteins between cells in an isogenic population). While we do not know what the molecular origin of this behavior is, we speculate this might reflect the cell-to-cell variations in lipid (tails') composition. Another point of interest here is the fact that the spread in our live *E. coli* data was greater at room temperature (23°C) than it was at growth temperature (37°C). This suggests that some cells may respond more readily to the temperature shift than others and this result further emphasizes the heterogeneity of the cell population.

A further noteworthy observation from Table 1 is the large difference between the viscosities measured in live *E. coli* membranes (both for healthy cells and spheroplasts) and those observed in *E. coli* extracts. Interestingly, the viscosity within liposomes of the membrane extracts was 200 cP at 37°C, and this is in good agreement with the value of circa 180–200 cP obtained previously for such extracts using EPR (10). This inconsistency between live cells and membrane extracts (liposomes) can be rationalized by considering the large differences that exist in the membrane environments of cells and extracts. Unlike liposomes made up of extracts, *E. coli* plasma membranes contain a high concentration of proteins (45), with up to 35% of the membrane area being occupied by proteins (46). Previous studies (45,47) have shown that diffusion coefficients of membrane proteins are 1–2 orders-of-magnitude higher in model membranes than they are in cells, which could explain the observed discrepancy. Additionally, the presence of the cytoskeleton in live cells may well also influence the effective membrane viscosity, as the cytoskeleton is currently thought to play a role in membrane organization (48). It has been reported that in the Gram-positive bacterium *B. subtilis* the cytoskeletal protein MreB creates specific membrane regions with increased fluidity (49). Single molecule fluorescence microscopy experiments have also suggested that the *E. coli* plasma membrane has domains with different physical properties (e.g., liquid-disordered regions) and that chemically induced depolymerization of MreB can influence the diffusion coefficient of fluorescent membrane probes (32). Yet another difference between the membranes of live *E. coli* cells and artificial membranes is that the former environment may display local heterogeneities and/or features of organization, which are likely lost when lipid extracts are reconstituted into artificial membranes. The organization of membranes of *E. coli* involves the separation of lipids, for example cardiolipin preferentially localizing to membrane poles (50–52) and the formation of zones enriched in either PE or PG (53). Membrane bilayer thickness, on the other hand, can be locally perturbed due to protein insertion causing a hydrophobic mismatch between proteins and lipids that has an impact on protein diffusion (45). All of the above effects will act to induce changes in the viscosities and diffusion coefficients observed in the membranes of liposomes and live cells and, as such, it is reasonable to expect that the values recorded in these two systems will differ considerably.

Finally, membrane viscosity can be linked to the diffusion coefficient using the Saffman-Delbrück approach (12), and calculated diffusion coefficients for the measured membrane viscosities of *E. coli* cells, spheroplasts, and *E. coli* lipid extracts can be seen in Table 2. While there are a number of limitations to the Saffman-Delbrück model (i.e., there are reports that this model fails for larger membrane proteins and for certain membrane viscosities (8,54,55)), it nonetheless remains a widely used approach and outlines the dependence of diffusion coefficients within membranes upon the viscosity. Applying this approach to the mean membrane viscosity values, we calculated the diffusion coefficient (for a molecule the size of BODIPY C10) within *E. coli* membranes to be 0.78 $\mu m^2 s^{-1}$ at 37°C and 0.63 $\mu m^2 s^{-1}$ at 23°C. These diffusion coefficients are comparable to those obtained by Nenninger et al. (24), who used the chemically similar BODIPY FL-C_{12} probe in live *E. coli* plasma membranes—1.2 $\mu m^2 s^{-1}$ and 0.6 $\mu m^2 s^{-1}$ at 37 and 23°C, respectively—and to the value of 1.5 $\mu m^2 s^{-1}$ reported by Oswald et al. (32).

The limitations of the Saffman-Delbrück approach highlight the importance of making direct measurements of fundamental parameters such as the viscosity, as reported here. In contrast to the research cited above, using FLIM of the molecular rotor BODIPY C10, we were able to obtain direct measurements of membrane viscosity that, crucially, are not reliant upon imperfect models of diffusion.

CONCLUSIONS

Fluorescence lifetime imaging of molecular rotors is a promising technique for the investigation of lipid bilayer viscosity, and, to the best of our knowledge, this represents the first direct measurement of bilayer viscosity within the plasma membranes of live *E. coli* bacteria. Using FLIM, we were able to directly determine the viscosity of live

E. coli plasma membranes and spheroplasts at both 23 and 37°C. In addition, we recorded membrane viscosities in liposomes consisting of *E. coli* lipid extracts over a range of temperatures. We showed that the plasma membrane of live *E. coli* cells displays a high viscosity of >900 cP, suggesting a high degree of lipid ordering within the liquid-phase membrane.

SUPPORTING MATERIAL

Nine figures and one table are available at http://www.biophysj.org/biophysj/supplemental/S0006-3495(16)30708-1.

AUTHOR CONTRIBUTIONS

Conceived the project, M.K.K. and J.T.M.; Designed the research, M.K.K., J.T.M., A.T., and M.R.D.; Performed experiments, J.T.M., A.T., and M.R.D.; Analyzed data, A.T. and M.R.D.; Contributed methods and materials: N.J.B., J.M., and J.H.; Wrote the paper: M.K.K., J.T.M., A.T., M.R.D., N.J.B., J.M., and J.H.

ACKNOWLEDGMENTS

The authors would like to express their gratitude to Prof. Anthony I. Magee, Dr Ursula Rodgers, Dr Antonio Konitsiotis, Mrs Mandy Hipwell and Prof. Bridget Leitinger for providing assistance in experiments and laboratory space to culture E. coli within the SAF building of Imperial College, London. J. T. M. acknowledges funding from NWO-Rubicon (project number 825.12.026) and a Marie Curie Actions Intra-European Fellowship (IEF, project number 329018). M. K. K. acknowledges the EPSRC for a Career Acceleration Fellowship (Grant EP/I003983/1). JH acknowledges financial support from the KU Leuven Research Fund (GOA2011/03, IDO/13/008), the Flemish government through long term structural funding Methusalem (CASAS2, Meth/15/04), the Hercules foundation (HER/08/021, HER/11/14) and the Belgian Federal Science Policy Office (IAP-VI/27).

REFERENCES

1. Gierasch, L. M., and A. Gershenson. 2009. Post-reductionist protein science, or putting Humpty Dumpty back together again. *Nat. Chem. Biol.* 5:774–777.
2. Gershenson, A., and L. M. Gierasch. 2011. Protein folding in the cell: challenges and progress. *Curr. Opin. Struct. Biol.* 21:32–41.
3. Cohen, J. E. 2004. Mathematics is biology's next microscope, only better; biology is mathematics' next physics, only better. *PLoS Biol.* 2:e439.
4. Mogilner, A., R. Wollman, and W. F. Marshall. 2006. Quantitative modeling in cell biology: what is it good for? *Dev. Cell.* 11:279–287.
5. Moran, U., R. Phillips, and R. Milo. 2010. SnapShot: key numbers in biology. *Cell.* 141:1262.e1.
6. Cowan, A. E., E. M. Olivastro, ..., P. Setlow. 2004. Lipids in the inner membrane of dormant spores of *Bacillus* species are largely immobile. *Proc. Natl. Acad. Sci. USA.* 101:7733–7738.
7. Zhang, Y.-M., and C. O. Rock. 2008. Membrane lipid homeostasis in bacteria. *Nat. Rev. Microbiol.* 6:222–233.
8. Hughes, B. D., B. A. Pailthorpe, and L. R. White. 1981. The translational and rotational drag on a cylinder moving in a membrane. *J. Fluid Mech.* 110:349–372.
9. Marr, A. G., and J. L. Ingraham. 1962. Effect of temperature on the composition of the fatty acids in *Escherichia coli*. *J. Bacteriol.* 84:1260–1267.
10. Sinensky, M. 1974. Homeoviscous adaptation—a homeostatic process that regulates the viscosity of membrane lipids in *Escherichia coli*. *Proc. Natl. Acad. Sci. USA.* 71:522–525.
11. Zhang, G., I. Fedyunin, ..., Z. Ignatova. 2010. Global and local depletion of ternary complex limits translational elongation. *Nucleic Acids Res.* 38:4778–4787.
12. Saffman, P. G., and M. Delbrück. 1975. Brownian motion in biological membranes. *Proc. Natl. Acad. Sci. USA.* 72:3111–3113.
13. Mika, J. T., and B. Poolman. 2011. Macromolecule diffusion and confinement in prokaryotic cells. *Curr. Opin. Biotechnol.* 22:117–126.
14. Dennis, W. H., and M. B. Yatvin. 1981. Correlation of hyperthermic sensitivity and membrane microviscosity in *E. coli* K1060. *Int. J. Radiat. Biol. Relat. Stud. Phys. Chem. Med.* 39:265–271.
15. Zaritsky, A., A. H. Parola, ..., H. Masalha. 1985. Homeoviscous adaptation, growth rate, and morphogenesis in bacteria. *Biophys. J.* 48:337–339.
16. Parola, A. H., M. Ibdah, ..., A. Zaritsky. 1990. Deviation from homeoviscous adaptation in *Escherichia coli* membranes. *Biophys. J.* 57:621–626.
17. Loison, P., N. A. Hosny, ..., J.-M. Perrier-Cornet. 2013. Direct investigation of viscosity of an atypical inner membrane of *Bacillus* spores: a molecular rotor/FLIM study. *Biochim. Biophys. Acta.* 1828:2436–2443.
18. Loison, P., P. Gervais, ..., M. K. Kuimova. 2016. Effect of ethanol perturbation on viscosity and permeability of an inner membrane in *Bacillus subtilis* spores. *Biochim. Biophys. Acta.* 1858:2060–2069.
19. Wu, Y., M. Stefl, ..., M. K. Kuimova. 2013. Molecular rheometry: direct determination of viscosity in Lo and Ld lipid phases via fluorescence lifetime imaging. *Phys. Chem. Chem. Phys.* 15:14986–14993.
20. López-Duarte, I., T. T. Vu, ..., M. K. Kuimova. 2014. A molecular rotor for measuring viscosity in plasma membranes of live cells. *Chem. Commun. (Camb.).* 50:5282–5284.
21. Kuimova, M. K., G. Yahioglu, ..., K. Suhling. 2008. Molecular rotor measures viscosity of live cells via fluorescence lifetime imaging. *J. Am. Chem. Soc.* 130:6672–6673.
22. Kuimova, M. K. 2012. Mapping viscosity in cells using molecular rotors. *Phys. Chem. Chem. Phys.* 14:12671–12686.
23. Mika, J. T., G. van den Bogaart, ..., B. Poolman. 2010. Molecular sieving properties of the cytoplasm of *Escherichia coli* and consequences of osmotic stress. *Mol. Microbiol.* 77:200–207.
24. Nenninger, A., G. Mastroianni, ..., C. W. Mullineaux. 2014. Independent mobility of proteins and lipids in the plasma membrane of *Escherichia coli*. *Mol. Microbiol.* 92:1142–1153.
25. Barthmes, M., M. D. F. Jose, ..., A. Koçer. 2014. Studying mechanosensitive ion channels with an automated patch clamp. *Eur. Biophys. J.* 43:97–104.
26. Warren, S. C., A. Margineanu, ..., P. M. W. French. 2013. Rapid global fitting of large fluorescence lifetime imaging microscopy datasets. *PLoS One.* 8:e70687.
27. Dent, M. R., I. López-Duarte, ..., M. K. Kuimova. 2015. Imaging phase separation in model lipid membranes through the use of BODIPY based molecular rotors. *Phys. Chem. Chem. Phys.* 17:18393–18402.
28. Hosny, N. A., C. Fitzgerald, ..., M. K. Kuimova. 2016. Direct imaging of changes in aerosol particle viscosity upon hydration and chemical aging. *Chem. Sci. (Camb.).* 7:1357–1367.
29. Hosny, N. A., G. Mohamedi, ..., M. K. Kuimova. 2013. Mapping microbubble viscosity using fluorescence lifetime imaging of molecular rotors. *Proc. Natl. Acad. Sci. USA.* 110:9225–9230.
30. Morton, L. A., J. P. Saludes, and H. Yin. 2012. Constant pressure-controlled extrusion method for the preparation of nano-sized lipid vesicles. *J. Vis. Exp.* 64:4151.

31. Mitra, K., I. Ubarretxena-Belandia, …, D. M. Engelman. 2004. Modulation of the bilayer thickness of exocytic pathway membranes by membrane proteins rather than cholesterol. *Proc. Natl. Acad. Sci. USA*. 101:4083–4088.

32. Oswald, F., A. Varadarajan, …, Y. J. M. Bollen. 2016. MreB-dependent organization of the *E. coli* cytoplasmic membrane controls membrane protein diffusion. *Biophys. J.* 110:1139–1149.

33. Wood, J. M. 2011. Bacterial osmoregulation: a paradigm for the study of cellular homeostasis. *Annu. Rev. Microbiol.* 65:215–238.

34. Konopka, M. C., I. A. Shkel, …, J. C. Weisshaar. 2006. Crowding and confinement effects on protein diffusion in vivo. *J. Bacteriol.* 188:6115–6123.

35. Pilizota, T., and J. W. Shaevitz. 2012. Fast, multiphase volume adaptation to hyperosmotic shock by *Escherichia coli*. *PLoS One*. 7:e35205.

36. Vysniauskas, A., M. Qurashi, …, M. K. Kuimova. 2015. Unravelling the effect of temperature on viscosity-sensitive fluorescent molecular rotors. *Chem. Sci. (Camb.).* 6:5773–5778.

37. Booth, I. R. 2002. Stress and the single cell: intrapopulation diversity is a mechanism to ensure survival upon exposure to stress. *Int. J. Food Microbiol.* 78:19–30.

38. Aertsen, A., and C. W. Michiels. 2005. Diversify or die: generation of diversity in response to stress. *Crit. Rev. Microbiol.* 31:69–78.

39. Mika, J. T., P. E. Schavemaker, …, B. Poolman. 2014. Impact of osmotic stress on protein diffusion in *Lactococcus lactis*. *Mol. Microbiol.* 94:857–870.

40. Dowhan, W. 1997. Molecular basis for membrane phospholipid diversity: why are there so many lipids? *Annu. Rev. Biochem.* 66:199–232.

41. Oursel, D., C. Loutelier-Bourhis, …, C. M. Lange. 2007. Lipid composition of membranes of *Escherichia coli* by liquid chromatography/tandem mass spectrometry using negative electrospray ionization. *Rapid Commun. Mass Spectrom.* 21:1721–1728.

42. Seu, K. J., L. R. Cambrea, …, J. S. Hovis. 2006. Influence of lipid chemistry on membrane fluidity: tail and headgroup interactions. *Biophys. J.* 91:3727–3735.

43. Ramadurai, S., R. Duurkens, …, B. Poolman. 2010. Lateral diffusion of membrane proteins: consequences of hydrophobic mismatch and lipid composition. *Biophys. J.* 99:1482–1489.

44. De Vries, A. H., A. E. Mark, and S. J. Marrink. 2004. The binary mixing behavior of phospholipids in a bilayer: a molecular dynamics study. *J. Phys. Chem. B.* 108:2454–2463.

45. Ramadurai, S., A. Holt, …, B. Poolman. 2009. Lateral diffusion of membrane proteins. *J. Am. Chem. Soc.* 131:12650–12656.

46. Dupuy, A. D., and D. M. Engelman. 2008. Protein area occupancy at the center of the red blood cell membrane. *Proc. Natl. Acad. Sci. USA*. 105:2848–2852.

47. Kumar, M., M. S. Mommer, and V. Sourjik. 2010. Mobility of cytoplasmic, membrane, and DNA-binding proteins in *Escherichia coli*. *Biophys. J.* 98:552–559.

48. Mueller, V., C. Ringemann, …, C. Eggeling. 2011. STED nanoscopy reveals molecular details of cholesterol- and cytoskeleton-modulated lipid interactions in living cells. *Biophys. J.* 101:1651–1660.

49. Strahl, H., F. Bürmann, and L. W. Hamoen. 2014. The actin homologue MreB organizes the bacterial cell membrane. *Nat. Commun.* 5:3442.

50. Mileykovskaya, E., and W. Dowhan. 2000. Visualization of phospholipid domains in *Escherichia coli* by using the cardiolipin-specific fluorescent dye 10-N-nonyl acridine orange. *J. Bacteriol.* 182:1172–1175.

51. Romantsov, T., S. Helbig, …, J. M. Wood. 2007. Cardiolipin promotes polar localization of osmosensory transporter ProP in *Escherichia coli*. *Mol. Microbiol.* 64:1455–1465.

52. Renner, L. D., and D. B. Weibel. 2011. Cardiolipin microdomains localize to negatively curved regions of *Escherichia coli* membranes. *Proc. Natl. Acad. Sci. USA*. 108:6264–6269.

53. Vanounou, S., A. H. Parola, and I. Fishov. 2003. Phosphatidylethanolamine and phosphatidylglycerol are segregated into different domains in bacterial membrane. A study with pyrene-labelled phospholipids. *Mol. Microbiol.* 49:1067–1079.

54. Guigas, G., and M. Weiss. 2006. Size-dependent diffusion of membrane inclusions. *Biophys. J.* 91:2393–2398.

55. Petrov, E. P., and P. Schwille. 2008. Translational diffusion in lipid membranes beyond the Saffman-Delbruck approximation. *Biophys. J.* 94:L41–L43.

Biophysical Journal, Volume *111*

Supplemental Information

Measuring the Viscosity of the *Escherichia coli* Plasma Membrane Using Molecular Rotors

Jacek T. Mika, Alexander J. Thompson, Michael R. Dent, Nicholas J. Brooks, Jan Michiels, Johan Hofkens, and Marina K. Kuimova

SUPPLEMENTARY MATERIAL

Measuring the viscosity of the *Escherichia coli* plasma membrane using molecular rotors.

JTM[1,#,*], AJT[2,#], MRD[2], NJB[2], JM[3], JH[1], MKK[2,*]

[#] These authors contributed to this work equally.
*To whom correspondence should be addressed: MKK, JTM

Supplementary Figures

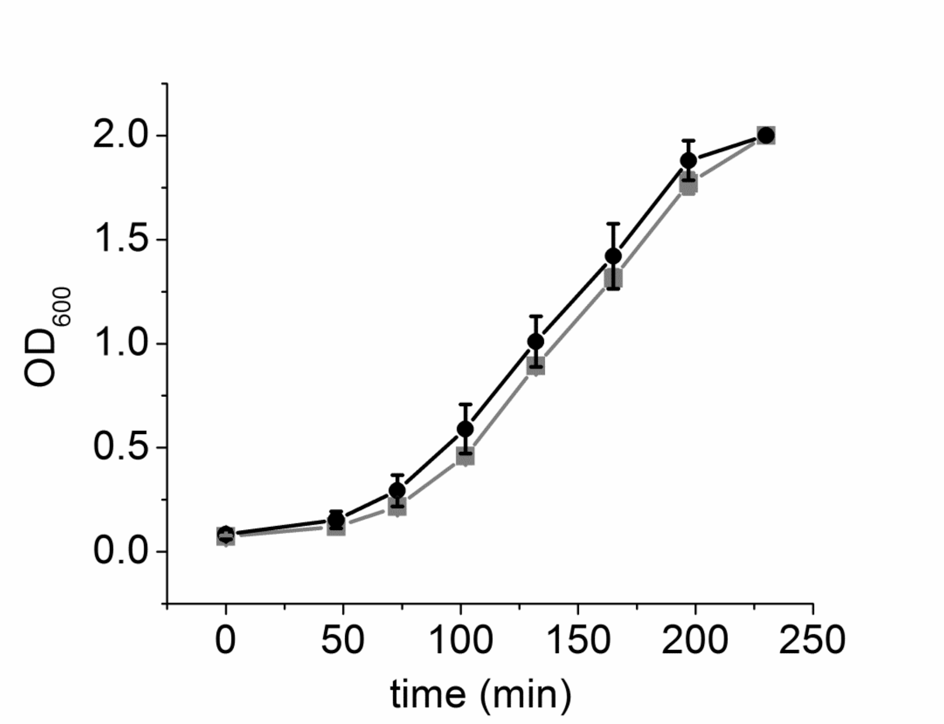

Figure S1. BODIPY C10 does not influence the growth of *Escherichia coli* at the concentrations used for fluorescence lifetime imaging.

E. coli cells were grown in LB medium at 37° C with vigorous shaking (200 rpm). A dense, overnight culture was used to inoculate fresh medium and this was allowed to grow until OD_{600} of 0.1 was reached. The cells grown in the presence of 0.2 μM BODIPY C10 (black circles) had very similar doubling times to cells grown without the dye (grey squares), as monitored by following the optical density of the cultures at 600 nm (OD_{600}). Error bars indicate the standard deviations from three measurements.

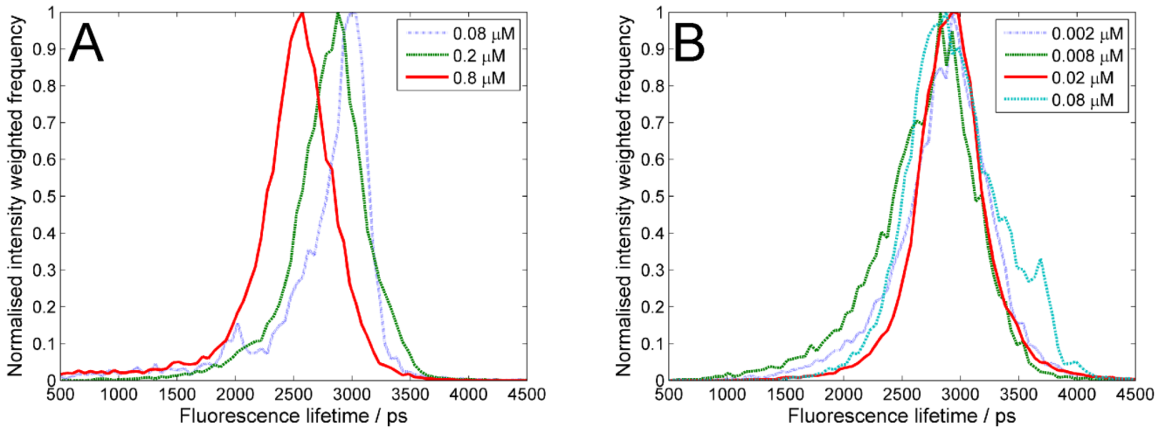

Figure S2. Optimisation of BODIPY C10 concentration to avoid aggregation.

Normalised intensity weighted fluorescence lifetime histograms recorded in populations of (A) healthy cells and (B) spheroplasts using a range of BODIPY C10 concentrations (see keys). Histograms were obtained from images fitted using a mono-exponential decay model (e.g. see Fig. 2). In spheroplasts (B), all lifetime distributions overlap indicating that dye aggregation has no adverse effects on the spheroplast data at any of the concentrations shown. In healthy cells (A), the lifetime distribution observed at a BODIPY C10 concentration of 0.8 μM is lower than that recorded at both 0.2 μM and 0.08 μM. This indicates that aggregation is inducing a reduction in the lifetime of BODIPY C10 at this concentration and, hence, destroying the viscosity sensitivity. At the two lower concentrations (0.2 μM and 0.08 μM) the lifetime distributions in live cells overlap well – although the peaks do not perfectly coincide, the intensity weighted mean fluorescence lifetimes extracted from the two histograms are in agreement to within 50 ps (2740 ps at 0.08 μM and 2790 ps at 0.2 μM). This confirms that aggregation has no impact on the healthy cell data for concentrations at or below 0.2 μM.

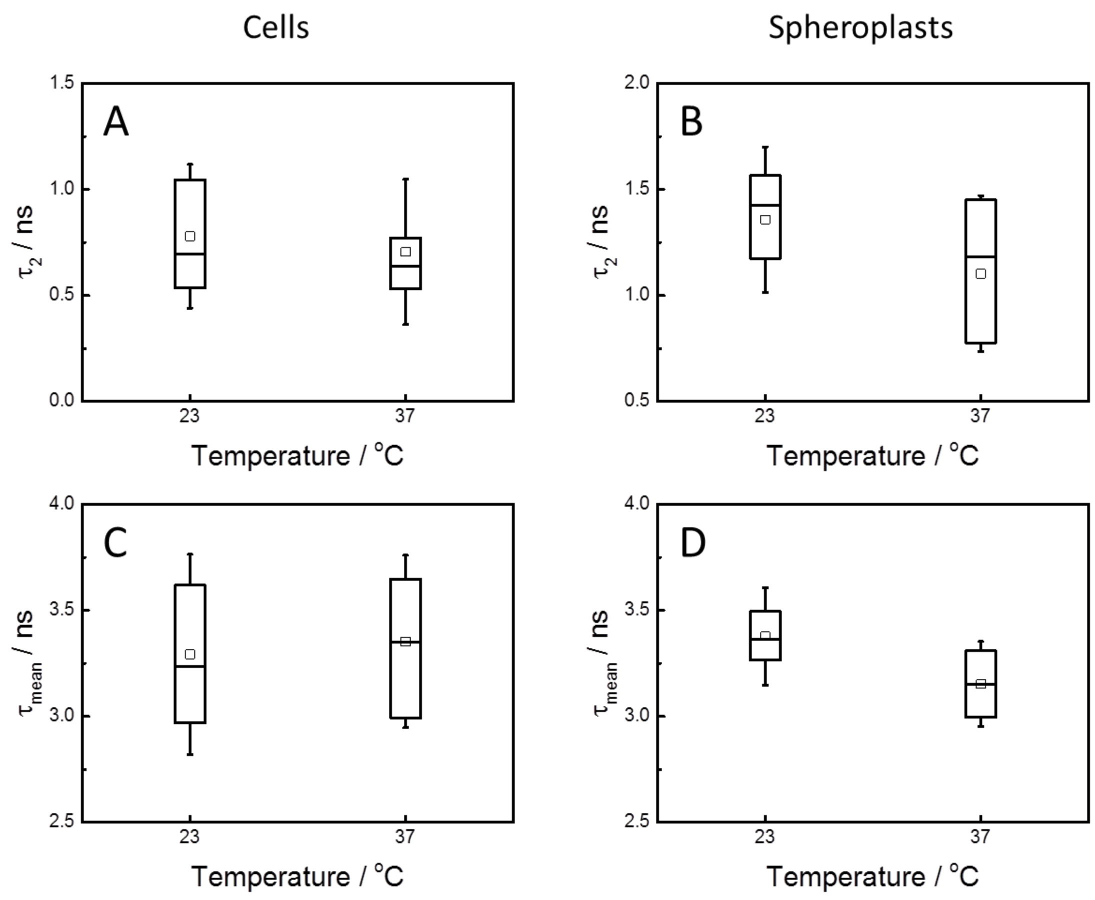

Figure S3. Behaviour of the additional fitted lifetime parameters (τ_2 and τ_{mean}) of BODIPY C10 in the membranes of live *E. coli* cells and spheroplasts.

Box plots showing the behaviour of τ_2 (top row – A, B) and τ_{mean} (bottom row – C, D) in healthy cells (left column – A, C) and spheroplasts (right column – B, D) at both 23° C and 37° C. Boxes represent the interquartile range; error bars (whiskers) indicate one standard deviation from the mean; horizontal lines show the median values; open squares indicate the mean values. Number of measurements: cells at 23° C – 21 cells from two independent experiments; cells at 37° C – 57 cells from four independent experiments; spheroplasts at 23° C – 28 spheroplasts from two independent experiments; spheroplasts at 37° C – 58 spheroplasts from four independent experiments.

Cells

	Lifetime (ns)		Viscosity (cP)	
	23° C	37° C	23° C	37° C
Mean	4.28	3.99	1160	950
SD	1.00	0.58	670	320
Median	4.00	3.78	960	830
1st Quartile	3.67	3.56	770	730
3rd Quartile	4.81	4.28	1410	1090
IQR	1.15	0.72	630	360

Spheroplasts

	Lifetime (ns)		Viscosity (cP)	
	23° C	37° C	23° C	37° C
Mean	4.45	4.06	1200	980
SD	0.42	0.42	240	210
Median	4.51	4.08	1220	980
1st Quartile	4.13	3.70	1010	790
3rd Quartile	4.74	4.43	1360	1170
IQR	0.61	0.73	350	380

Table S1. Statistics of the FLIM measurement of BODIPY C10 in a population of *Escherichia coli* cells and spheroplasts.

Table shows the lifetimes and viscosities observed in *E. coli* cells (top) and spheroplasts (bottom) at both 23° C and 37° C. SD – standard deviation, IQR – inter-quartile range. Number of measurements: n = 57 cells at 37° C; n = 21 cells at 23° C; n = 58 spheroplasts at 37° C; n = 28 spheroplasts at 23° C.

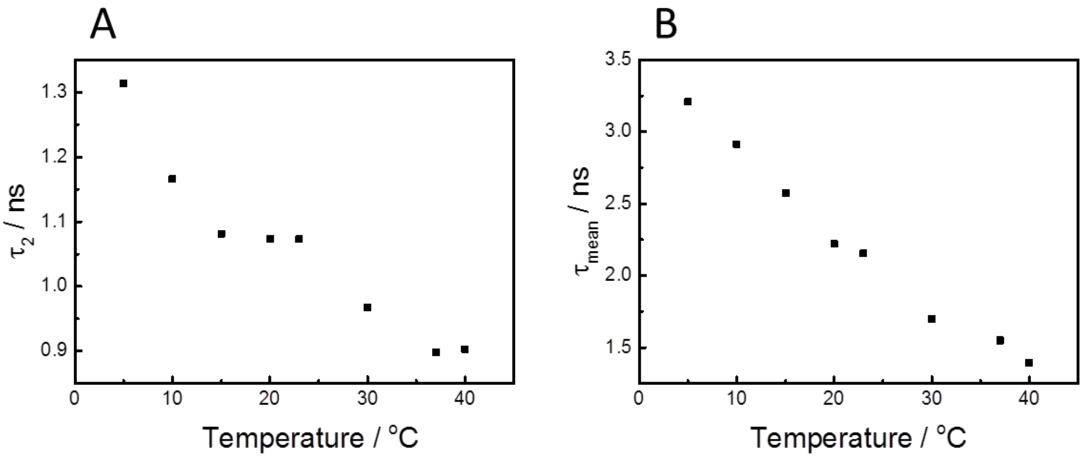

Figure S4. Behaviour of the additional fitted lifetime parameters (τ_2 and τ_{mean}) of BODIPY C10 in liposomes made of *E. coli* lipid extracts as functions of temperature.

TCSPC measurement of BODIPY C10 labelled liposomes composed of *E. coli* membrane lipid extracts, measured as a function of temperature. The behaviour of both the second (short) lifetime component (τ_2 – A) and the weighted mean lifetime (τ_{mean} – B) with respect to temperature are shown. Liposomes were prepared using lipid extracts from *E. coli* cells grown at 37° C.

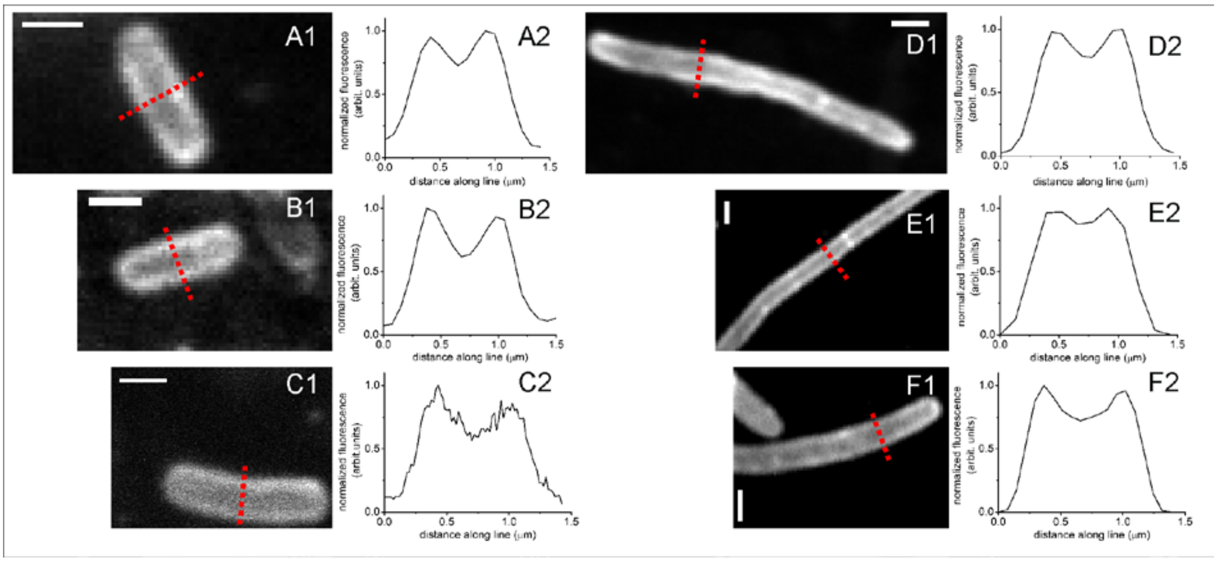

Figure S5. The molecular rotor BODIPY C10 stains membranes in live *Escherichia coli* cells.

Confocal fluorescence images of healthy (A1-C1) and cephalexin treated (D1-F1) *E. coli* cells stained with BODIPY C10. Scale bars 1 μm. Line profiles of fluorescence intensity were extracted along the red dotted lines shown in each image. The line profiles (subpanels with index 2, A2-F2) have two maxima that correspond to the locations of the membranes in the fluorescence images. Staining and fluorescence profiles of this sort indicate that the fluorescent probe BODIPY C10 is located within the membrane.

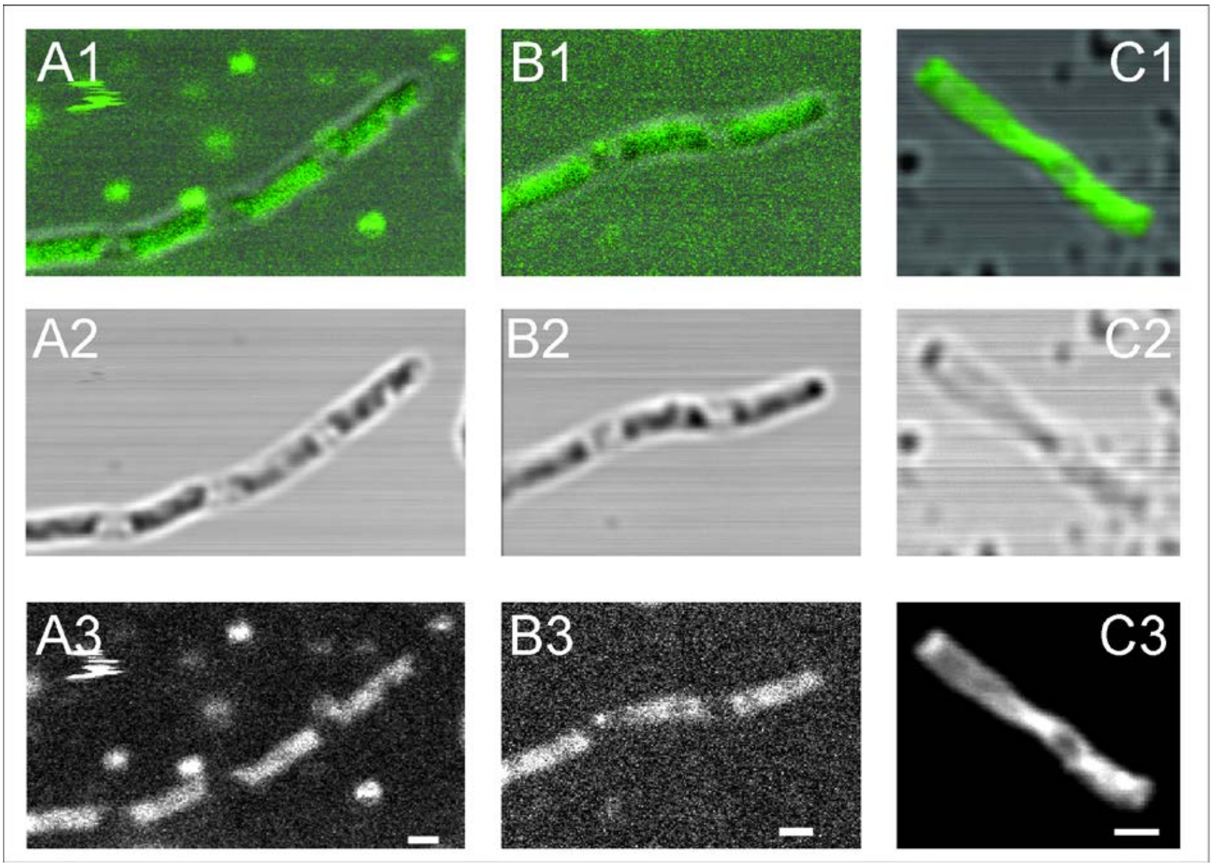

Figure S6. BODIPY C10 stains of cephalexin treated *Escherichia coli* cells subjected to osmotic shock.

In order to test whether the inner or outer membrane was stained by BODIPY C10, *E. coli* cells were stained with BODIPY C10 and were osmotically shocked by increasing the medium osmolality with 15% sucrose. In addition, to obtain elongated cells – in which the visual plasmolysis spaces (VPS, indicators of inner membrane staining) are readily visible – the cells were treated with cephalexin (see Materials and methods section). A1, B1 and C1 show overlays of transmittance (A2, B2 and C2) and fluorescence (A3, B3 and C3) images. Scale bars are 1 µm in length. The transmittance images (A2, B2, C2) indicate that cells have undergone osmotic shock. In the fluorescence images (A3, B3, C3) it can be observed that the stain no longer shows a rod like shape characteristic of cells under normal osmotic conditions (compare with Figure S5 and Fig. 1A-D). Instead the membranes have become invaginated. Moreover, VPS can be observed. This indicates that the rotor BODIPY C10 stains the inner (plasma) membranes of the cells.

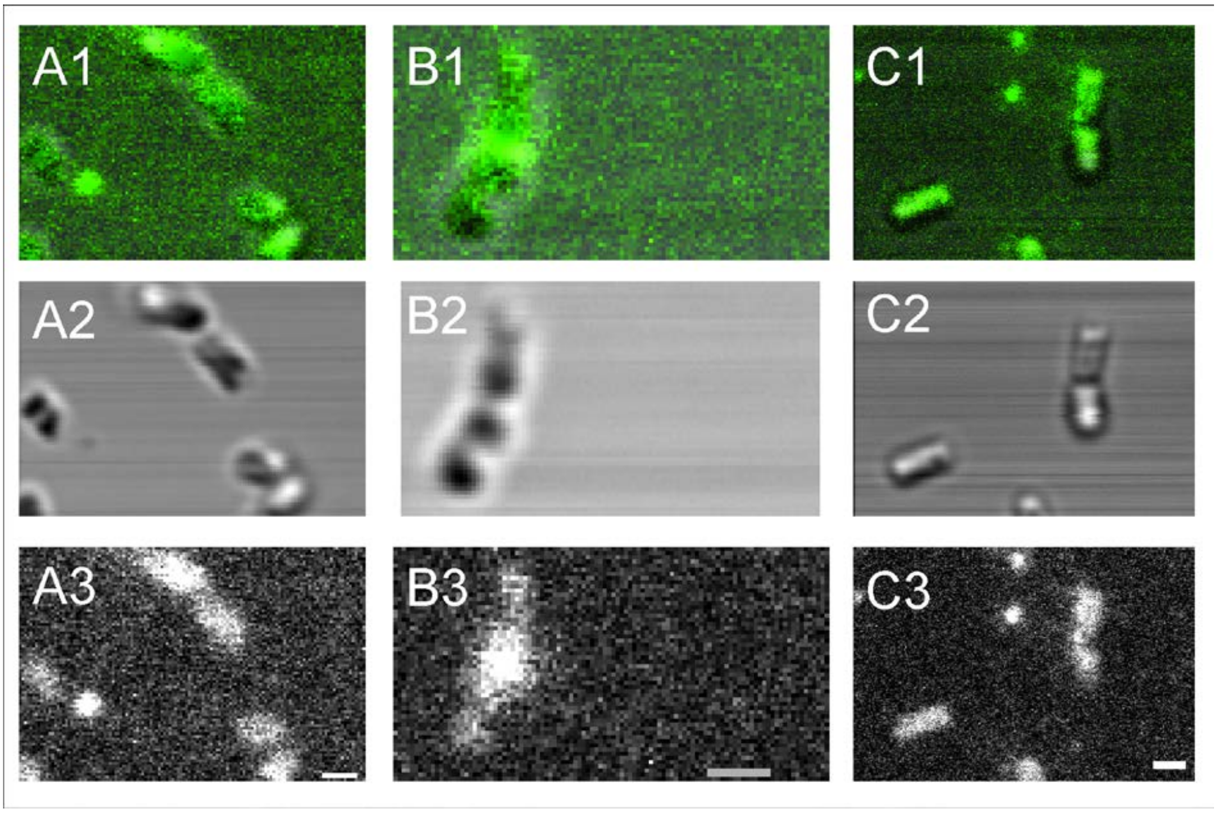

Figure S7. BODIPY C10 stains of healthy *Escherichia coli* cells subjected to osmotic shock.

E. coli cells were stained with the rotor BODIPY C10 and osmotically shocked by increasing medium osmolality with 15% sucrose. A1, B1 and C1 show overlays of transmittance (A2, B2, C2) and fluorescence (A3, B3, C3) images. Scale bars 1 µm. The transmittance images (A2, B2, C2) clearly indicate that cells have undergone osmotic shock. In the fluorescence images (A3, B3, C3) it can be observed that the stain no longer shows a rod like shape characteristic of cells under normal osmotic conditions (compare with Figure S5 and Fig. 1A-D). Instead the membranes appear invaginated and perturbed. This indicates that the rotor BODIPY C10 stains the inner (plasma) membranes of the cells.

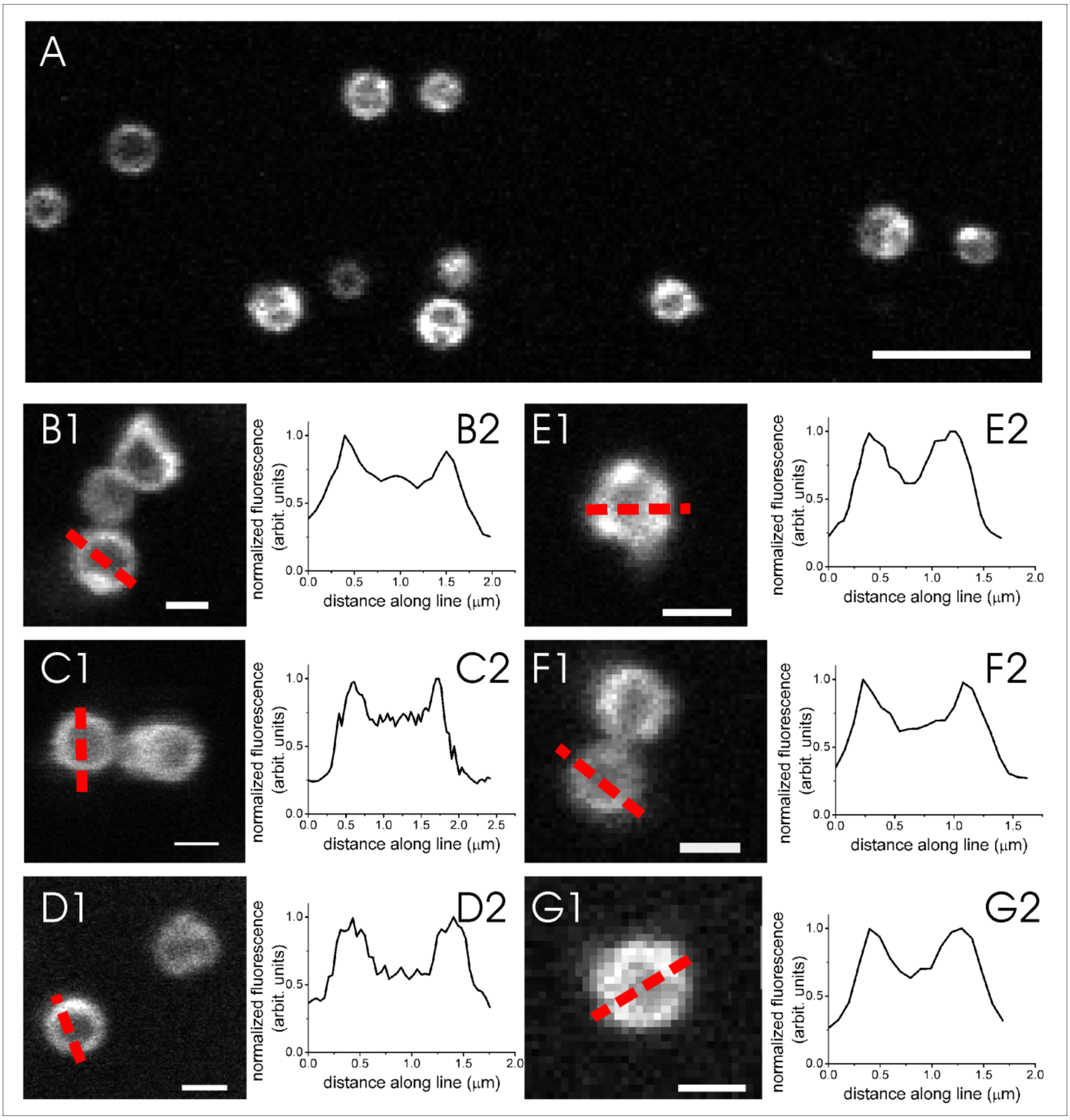

Figure S8. The molecular rotor BODIPY C10 stains membranes in *Escherichia coli* spheroplasts.

Confocal fluorescence images of spheroplasts generated from live *E. coli* cells stained with BODIPY C10. (A) Image showing the membrane stain obtained with BODIPY C10 in several spheroplasts. Scale bar 5 μm. (B-G) Examples of individual spheroplasts stained with BODIPY C10, where a line profile of fluorescence intensity was extracted along the red dotted line. Scale bars 1 μm. The line profiles (subpanels with index 2) have two maxima that correspond to the locations of the membranes in the diffraction limited images. Staining and fluorescence profiles of this sort are indicative of a membrane localised probe.

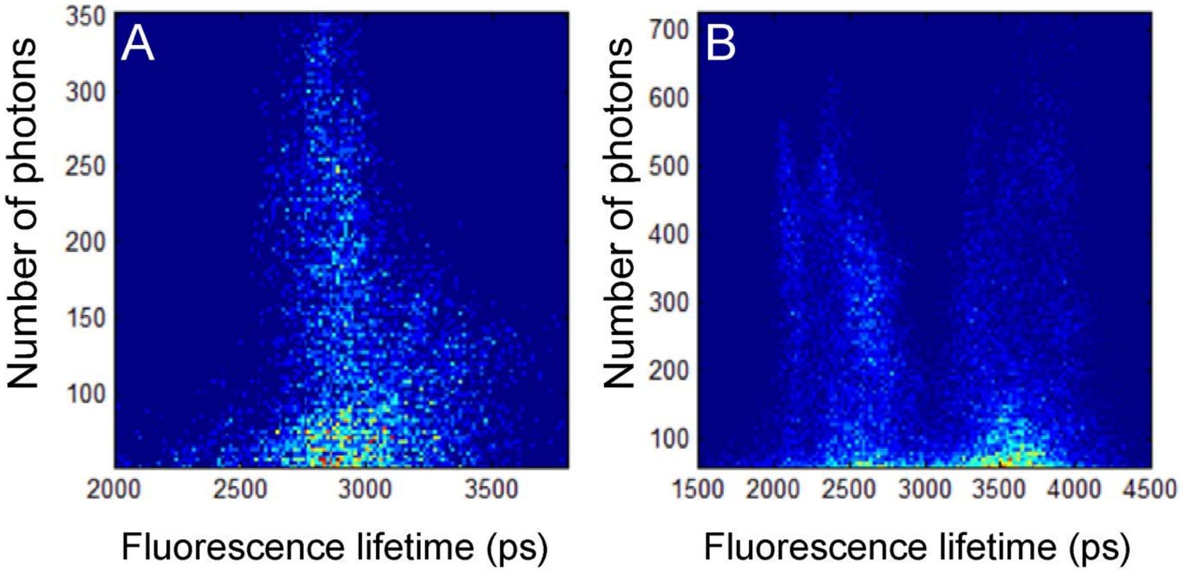

Figure S9. Correlation of fluorescence lifetime and intensity in BODIPY C10 stained live *Escherichia coli* cells.

Correlation of the fluorescence intensity and the fluorescence lifetime for two representative FLIM images (generated using mono-exponential fitting) of *E. coli* stained with BODIPY C10. (A) Cells grown at 37° C and imaged at 23° C; (B) cells grown and imaged at 37° C. The false colour scale represents the number of pixels with a given lifetime and intensity (red – high, blue – low). In both cases the lifetime is observed to be approximately invariant as the intensity increases. This is even the case in (B), where a bimodal lifetime distribution is observed. Thus, fluorescence intensity and lifetime are uncorrelated.
